# Identification and Validation of Cyclic Peptides with Mucin-Selective, Location-Specific Binding in the Gastrointestinal Tract

**DOI:** 10.1021/acsnano.4c13520

**Published:** 2025-04-11

**Authors:** Deepak A. Subramanian, Austin Chin, Yunhua Shi, Gary W. Liu, Robert Langer, Giovanni Traverso

**Affiliations:** † Department of Chemical Engineering, Massachusetts Institute of Technology, Cambridge, Massachusetts 02139, United States; ‡ David H. Koch Institute for Integrative Cancer Research, Massachusetts Institute of Technology, Cambridge, Massachusetts 02139, United States; § Department of Mechanical Engineering, Massachusetts Institute of Technology, Cambridge, Massachusetts 02139, United States; ∥ Division of Gastroenterology, Brigham and Women’s Hospital, Harvard Medical School, Boston, Massachusetts 02115, United States

**Keywords:** peptide, mucin, binding, specificity, gastrointestinal, localization

## Abstract

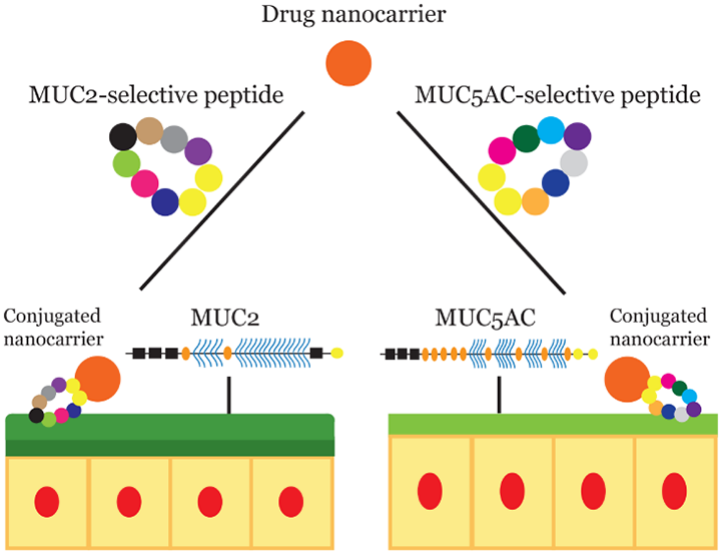

Oral drug delivery is a widely preferred method of drug administration due to its ease of use and convenience for patients. Localization of drug release in the gastrointestinal (GI) tract is important to treat localized diseases and maximize drug absorption. However, achieving drug localization in the dynamic GI tract is challenging. To address this challenge, we leveraged the geographic diversity of the GI tract by targeting its mucus layers, which coat the epithelial surfaces. These layers, composed of mucin glycoproteins, are synthesized with unique chemical compositions and expressed in different regions, making them ideal targets for drug localization. In this article, we identify cyclic peptides that bind selectively to MUC2 (in the intestines) and MUC5AC (in the stomach), serving as targeting ligands to these regions of the GI tract. We demonstrate the effectiveness of these peptides through *in vitro*, *ex vivo*, and *in vivo* experiments, showing that incorporating these targeting ligands can increase binding and selectivity 2-fold to the desired regions, thus potentially overcoming challenges with localizing drug distribution in oral delivery. These results indicate that cyclic peptides can be used to localize drug cargoes at certain sites in the body compared to free drugs.

Oral drug delivery is preferred for drug administration because it is less invasive, less painful, and easier to administer compared to parenteral drug delivery.^1^ This approach enhances treatment efficacy by improving patient adherence to the drug therapy regimen.^2^ However, the gastrointestinal (GI) tract presents several barriers to successful drug delivery, such as the hostile luminal environment with degrading enzymes, bile salts, and food (particularly for macromolecular and therapeutic bacterial cargoes),^3^ and poor gastrointestinal retention.^4^ Another potential issue is that the therapeutic efficacy of certain drugs is reliant on their location within the gastrointestinal tract, meaning that ineffective drug targeting can lead to inefficient treatment.^5^

Targeted drug delivery involves ways of controlling drug localization and release to a certain area of the body based on various desired outcomes.^6^ Some potential motivations for targeted drug delivery within the gastrointestinal tract include controlling the release of a drug at a specific location to treat localized diseases (such as stomach ulcers,^7^ inflammatory bowel disease,^8^ and colorectal cancer),^9^ improving the absorption of drugs and therapeutic peptides by localizing their release at the optimal absorption location (such as aspirin in the stomach^10^ and insulin in the colon^11,12^) and increasing drug residency by “anchoring” to specific regions of the body.^13,14^

Materials can be selected to enhance drug delivery by improving drug localization and extending GI transit time.^15^ In this area, orally administered nanoparticles have been widely studied due to their ability to optimize release kinetics and protect the drug cargo from the degradative environments in the GI tract.^16^ One effective method involves mucoadhesion;^17^ for example, nanoparticles for oral delivery of macromolecules, composed of mucoadhesive materials, such as alginate, chitosan, and poly(acrylic acid), among others^15^ have been engineered to bind to the mucus layers covering the epithelial surfaces of the GI tract, enabling greater retention time in a specific location for drug release compared to unmodified drugs.^18^ Mucoadhesive approaches have been demonstrated with various nanoparticles and encapsulated drugs;^19^ however, these approaches typically rely on nonspecific binding to mucus through electrostatic, wetting, and mechanical interlocking interactions with the negatively charged mucus.^18,20^ While this enhances the residence time in the GI tract, it does not facilitate targeting due to the ubiquitous presence of mucus throughout the GI tract, which may lead to drug release in suboptimal locations for absorption. Additionally, the general mucoadhesive approach is less effective for treating location-specific diseases, such as ulcerative colitis in the small intestine/colon or ulcers in the stomach, due to its poor targeting capability.

Peptides have been investigated as potential targeting ligands due to their enhanced specificity when binding to targeted structural pockets while also being small enough to fit snugly into smaller binding pockets. This is in contrast to antibodies, which also have very strong specificity but are relatively large.^21^ Peptides have been used to bind to various targets in the GI tract, such as goblet cells,^22^ integrin receptors,^23^ thrombin receptors,^24^ pancreatic β cells,^25^ M cells,^26^ and Peyer’s patches.^27^ Despite their advantages, the largest challenge with using peptide ligands for orally administered drug carriers is degradation in the digestive tract. The harsh stomach environment, with a low pH (∼p1.6–2), can disrupt the secondary and tertiary structures of peptides. Proteases and other digestive enzymes can cleave peptide structures, both of which reduce peptide targeting efficacy.^28^ The small intestine also contains a high density of digestive enzymes, further reducing peptide stability during oral administration.^29^

One of the most commonly used methods for peptide stabilization is the use of cyclic peptides. Cyclic peptides, which create a “loop-like” structure through intrapeptide bonds between amino acid residues, such as lactam, lactone, and disulfide bonds, as well as synthetic macrocyclic peptides, which contain rings with at least 10 amino acid residues, have been shown to resist protease activity^30^ and maintain peptide structure in the gastrointestinal environment. One of the most commonly used methods is the formation of disulfide bonds between nonadjacent cysteine residues, creating cycles of various sizes.^31^ This method can also generate multicyclic structures by including multiple sets of paired cysteine residues.^32^ Disulfide bond cyclization has been shown to improve both resistance to protease activity^33^ and target affinity and selectivity,^34^ ultimately leading to greater therapeutic activity.^35^ In addition, libraries with disulfide-bound cyclic peptides are relatively easy to screen and use due to the existence of commercially available phage display libraries containing these peptides.

Recognizing the differences in mucus structure and composition throughout the GI tract, we hypothesized that the mucin glycoproteins (which compose the mucus layers) could be leveraged as binding targets to impart specificity. The mucus layers in the small intestine and colon are primarily composed of the mucin MUC2,^36^ those in the stomach mainly contain the mucins MUC5AC and MUC6,^37^ and the mucus layers in the salivary glands and esophagus predominantly consist of the mucin MUC5B.^38^ Despite structural similarities, such as D domains, cysteine-rich regions, and “PTS” domains, each mucin has a unique chemical structure, offering distinct targeting opportunities. For example, MUC2 features large PTS domains separated by two cysteine-rich domains, whereas MUC5AC is a larger glycoprotein with multiple cysteine-rich regions separating the PTS domains.^39^ The greater presence of cysteine-rich regions in MUC5AC compared with MUC2 highlights a chemical distinction that could be exploited to target one mucin over the other. As an example, we envision developing peptide-conjugated nanoparticles that can selectively target regions of the GI tract based on mucin expression (Figure 1).

**Figure 1:**
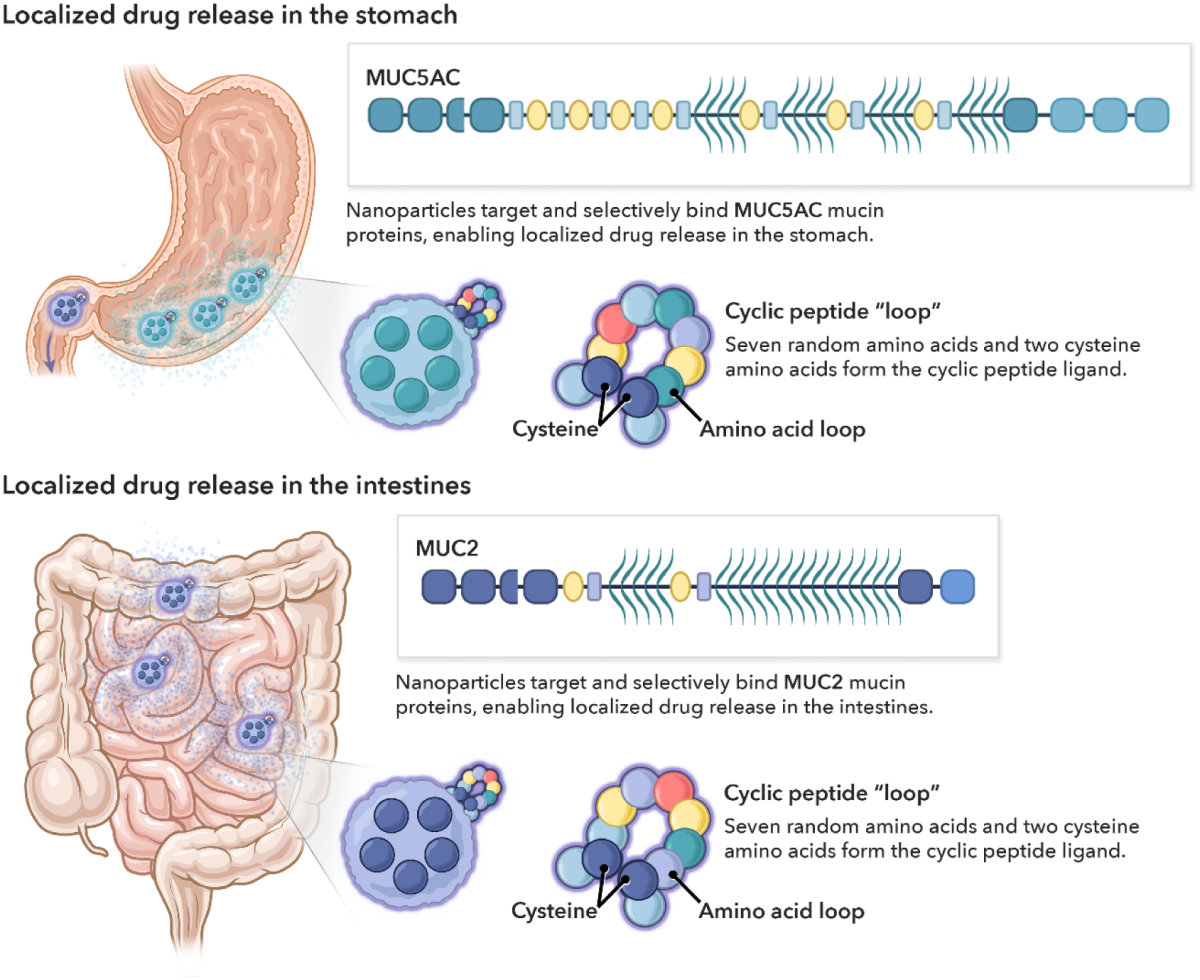
Schematic of one type of stomach-specific and intestine-specific drug delivery approach (peptide-conjugated nanoparticles) based on binding to the mucin glycoproteins found in these environments (MUC5AC in the stomach, MUC2 in the intestines). In this scenario, drug-loaded particles contain cyclic peptides conjugated to the surface, which confer targeting capability. The cyclic peptides are conjugated to the surface of the nanoparticle as shown in the figure, where the dark blue circles represent cysteine amino acids, and the other circles represent the amino acids that compose the cyclic peptide. The legend at the bottom shows which elements of the structure correspond to different regions of the mucins (D-domain, cysteine-rich region, PTS domain, and cysteine knot). Schematic of mucin structures adapted from Subramanian et al.^15^

In this work, we identify cyclic peptide-based ligands that can bind selectively to two different mucins: MUC2 and MUC5AC, to facilitate drug localization in the small intestine and stomach, respectively. We first use phage display to screen a large library of potential cyclic peptide hits (∼10^9^ unique sequences) in order to identify the motifs and sequences that indicate selective mucin binding. We then validated the performance of these hits in an *ex vivo* setting on freshly dissected porcine tissue to demonstrate their capability to bind to an intact mucus layer. We then measured the *in vitro* binding kinetics of these top hits to confirm the selective nature of binding. Finally, we show their capability to selectively localize and retain a model cargo *in vivo* in a rodent model.

## Results and Discussion

### Identified Peptides from Phage Display

**Figure 2:**
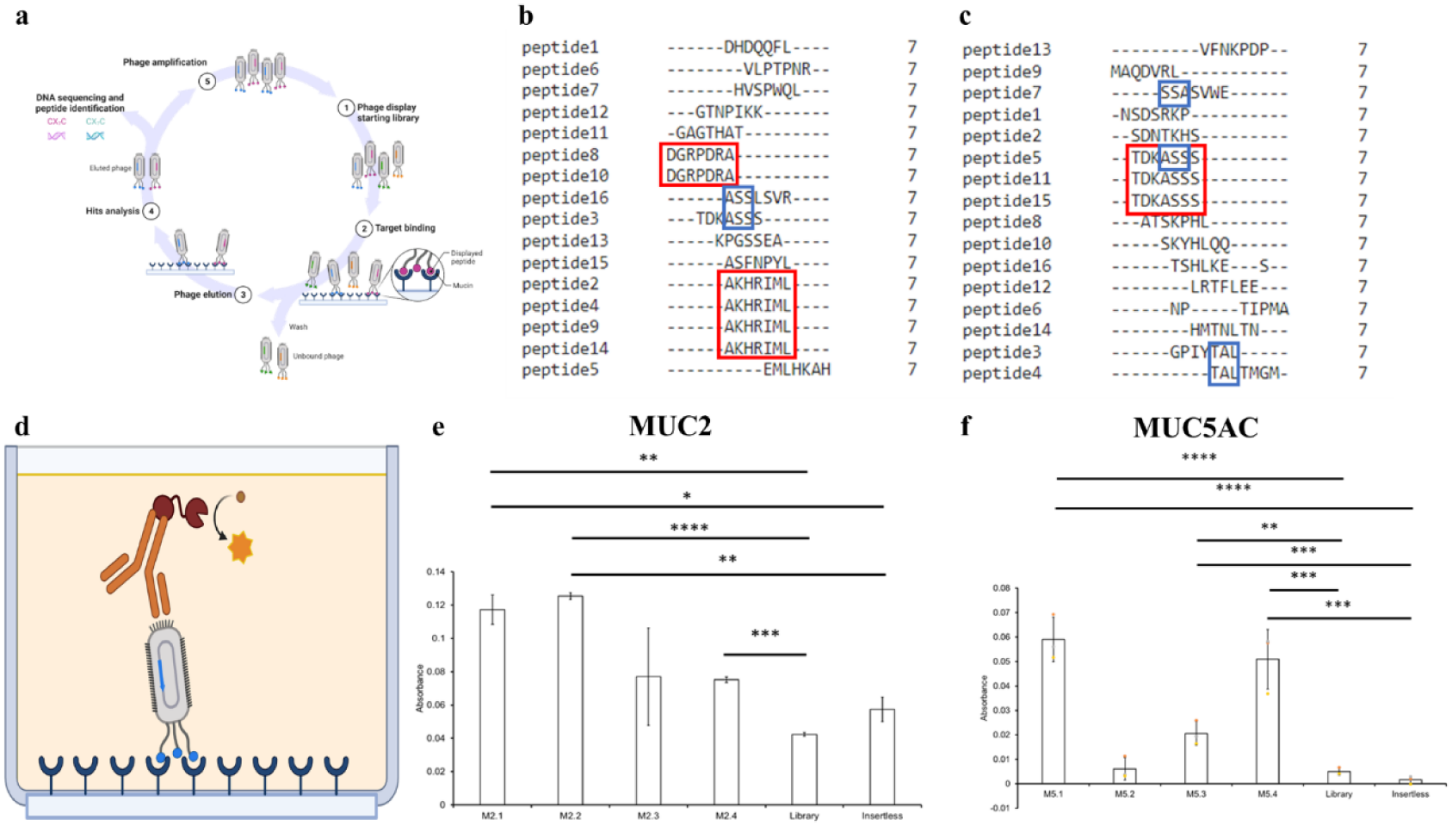
*In vitro* identification and validation of mucin-binding peptides. Peptides were identified from a large library of cyclic peptides using phage display (a), and peptides were identified for MUC2 (b) and MUC5AC (c); the sequences shown reflect the middle seven amino acids (X_7_) within the CX_7_C peptide structure to reflect the common sequences and motifs. To validate their performance, phage ELISA was employed (d), and the background-subtracted phage ELISA results were obtained for MUC2-binding hits (e) and MUC5AC-binding hits (f). In (b) and (c), red boxes represent common sequences, while blue boxes represent common motifs (or sequences of three or more amino acids that are shared among two distinct peptide sequences). In (e) and (f), statistical significance is conveyed by the following: *p < 0.05, **p < 0.01, ***p < 0.005, ****p < 0.001 (one-way ANOVA and post-hoc Bonferroni for multiple comparisons).

A cyclic CX_7_C peptide phage display was used to identify cyclic peptides that bind to MUC2 and MUC5AC. This process involves performing panning steps (incubation of library phage with the target of interest in Tris-buffered saline), followed by washing steps that remove phages that did not bind or bound weakly to MUC2/MUC5AC proteins. The remaining strongly bound phage were then amplified to create the starting population for the next cycle (Figure 2a). Sanger sequencing for phage display libraries screened on MUC2 (shown in Figure 2b) revealed 16 potential phage candidates. Across the phage candidates, the repeated peptide hits were CAKHRIMLC (4 of 16 candidates) and CDGRPDRAC (2 of 16 candidates). In addition, 2 of the 16 candidates exhibited the consensus motif “ASS”. Figure 2c shows the DNA sequencing results for phage display against MUC5AC. In this case, there was one repeated peptide sequence (CTDKASSSC, found in 3 out of 16 sequences) and two common motifs: SSA/ASS, found in 4 out of 16 sequences, and TAL, found in 2 out of 16 sequences. The phage clones that contained the repeated sequences and motifs were selected to progress to the validation stage; Table 1 shows the identities of the selected peptide sequences.

**Table 1:** Top Candidates from Phage Screening Against MUC2 and MUC5AC

Mucin Target	Peptide Sequence	Peptide ID	% Prevalence
MUC2	CAKHRIMLC	M2.1	25
MUC2	CTDKASSSC	M2.2	6.25
MUC2	CDGRPDRAC	M2.3	12.5
MUC2	CASSLSVRC	M2.4	6.25
MUC5AC	CTDKASSSC	M5.1	18.75
MUC5AC	CGPIYTALC	M5.2	6.25
MUC5AC	CTALTMGMC	M5.3	6.25
MUC5AC	CSSASVEWC	M5.4	6.25

### Validation of Phage Binding with Phage ELISA

We next sought to validate the binding efficiency of these identified phage clones. We tested phage clones displaying peptides M2.1, M2.2, M2.3, and M2.4 for binding to MUC2, and phage clones displaying peptides M5.1, M5.2, M5.3, and M5.4 for binding to MUC5AC. We utilized a bacteriophage enzyme-linked immunosorbent assay to test the binding of the peptide hits to the specific target mucins, MUC2 and MUC5AC (Figure 2d). The spectrophotometer reported absorbance values at 450 nm. The intensity of the absorbance signal is proportional to the amount of peptide-mucin binding present in each sample. The negative controls (library samples and insertless samples) served as references for nonspecific binding.

Figure 2e shows the phage ELISA results for the MUC2-selective peptides and controls, with the values for the blank samples subtracted from the other results. As shown below, M2.1, M2.2, and M2.4 exhibited significantly higher absorbances than the two negative controls (random library and insertless phage). Figure 2f shows the phage ELISA results for the MUC5AC-selective peptides and controls, with the values for the blank samples subtracted from the results for the hits and controls. M5.1, M5.3, and M5.4 exhibited significantly greater binding to MUC5AC than the random library and insertless phage. These results suggest that our phage display strategy was able to identify peptide sequences that exhibited binding to the intended MUC proteins.

### Selective Binding to Mucin-Containing Tissue *Ex Vivo* for Peptide Ligands

**Figure 3:**
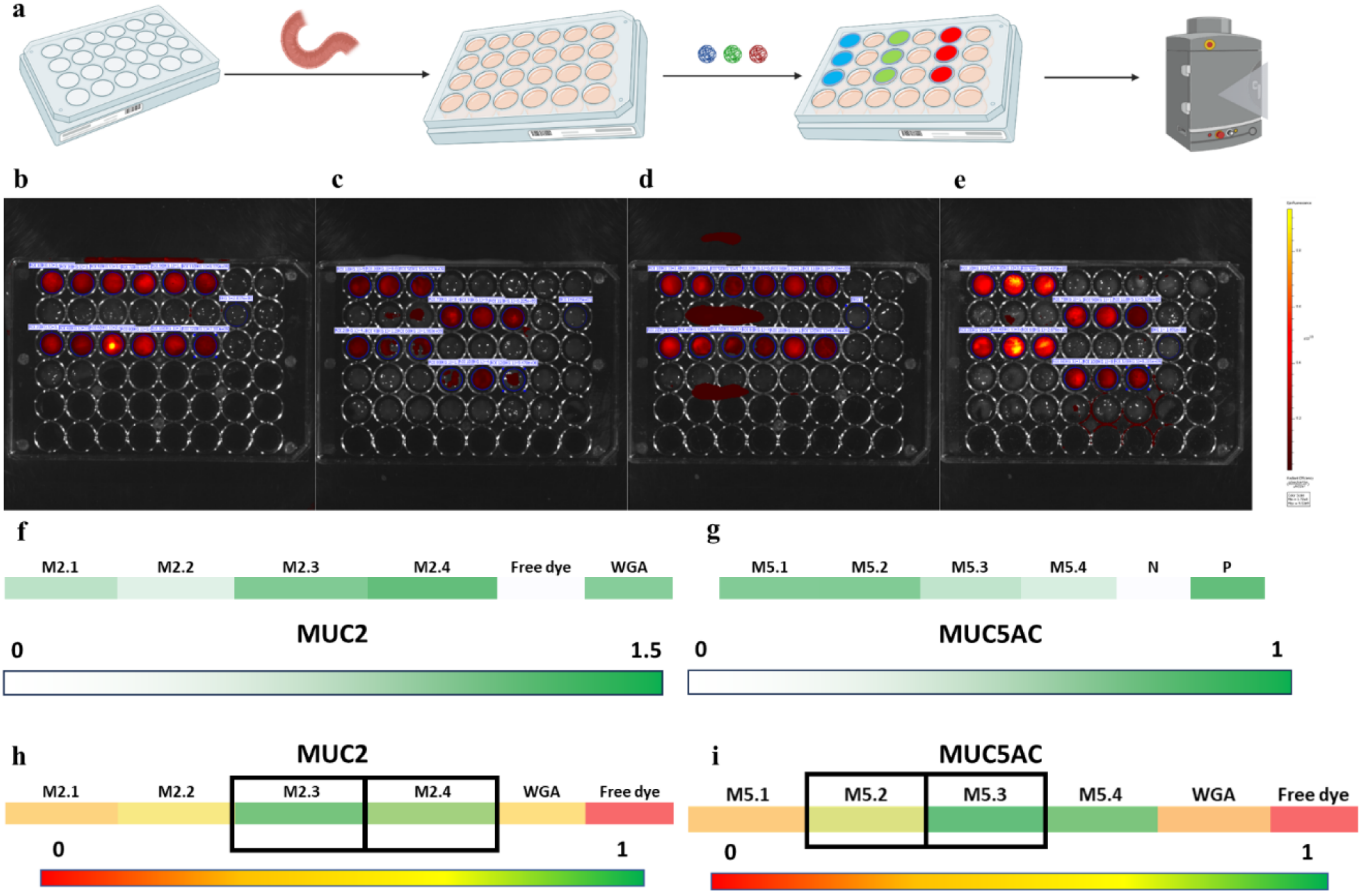
*Ex vivo* binding analysis for identified hits. The fluorescently tagged peptides were incubated with magnetically clamped pieces of mucin-containing tissue, and the fluorescence of the bound nanoparticles was semiquantitatively analyzed using *in vivo* imaging and spectroscopy (a). Representative IVIS images for measurement of the *ex vivo* binding of MUC2-selective peptides to the small intestine (b) and stomach (c) and MUC5AC-selective peptides to the small intestine (d) and stomach (e) are shown here. “Score” results indicating the average *ex vivo* binding magnitude are shown for the MUC2-binding (f) and MUC5AC-binding (g) hits to the tissue of interest (small intestine for MUC2-selective peptides and stomach for MUC5AC-selective peptides); the score is calculated by normalizing the individual fluorescence values against those of the negative and positive controls for each plate. Overall selectivity to the mucin of interest is shown for the MUC2-binding (h) and MUC5AC-binding (i) hits. The top hits are shown for each mucin with black boxes based on their binding strength and selectivity.

After identifying the peptide hits for each mucin, we tested their binding in an *ex vivo* setting (Figure S1), evaluating their interaction with mucin-containing porcine tissue and semiquantitatively representing it through fluorescent imaging (Figure 3a). The cyclic peptides were synthesized and conjugated to a fluorophore, Alexa Fluor 647 (AF647), at the *N*-terminus to allow fluorescent imaging to be used to semiquantitatively measure the peptides’ *ex vivo* tissue binding, compared to free AF647 and AF647 conjugated to wheat germ agglutinin (WGA), a known lectin for glycoproteins.^40^ A comparison of the raw fluorescence values for peptide-AF647 and WGA-AF647 showed similar fluorescence values at equal concentrations of modified dye, facilitating direct comparisons between the constructs (Figure S2). Porcine GI tissue was used due to its similarity in mucin expression compared to human GI tissue and its capacity to source large surface areas of tissue for experiments.^41,42^ Representative IVIS images are shown in Figure 3b-e, where the first four columns represent the four identified peptides (M2.1–M2.4 in Figure 3b,c, M5.1–M5.4 in Figure 3d,e), the fifth column represents WGA-AF647, and the sixth column represents AF647. The “score” results for the peptide ligands are presented in Figure 3f,g for the mucins of interest, with Tables S2–S5 and Figure S3 detailing results for the other tissues (the equation for calculating the “score” is found in the Materials and Methodssection). Despite some variability, the peptide hits generally exhibited stronger binding to tissues containing the mucin of interest (small intestine for MUC2-selective peptides and stomach for MUC5AC-selective peptides). We also examined selectivity by incubating peptides on nontarget tissues. Figure 3h,i depicts the “selectivity” results for the MUC2-selective and MUC5AC-selective peptides, respectively. Selectivity was calculated by determining the ratio of the “score” for a particular mucin to the sum of the “scores” for all mucins (see the *Materials and Methods* section for the equation used).

Specifically, M2.3 and M2.4 were shown to have high selectivity (>0.7) for MUC2-containing intestinal tissue and high fluorescence scores (>1), indicating that they bound to the MUC2-containing tissues more strongly and selectively than wheat germ agglutinin. M5.2 and M5.3 had moderate to high selectivity (>0.6) for MUC5AC-containing stomach tissue, as well as moderate to high fluorescence scores. Although M5.4 had a higher selectivity value than M5.2, the fluorescence score obtained through spectrophotometry showed low levels of binding, thus presenting M5.4 as an unfavorable peptide candidate.

To demonstrate its ability to localize cargo at longer time points *ex vivo*, additional studies were conducted to compare the binding of the peptide “hits” to mucin-containing and off-target tissues at 2, 4, and 8 h, comparing the peptides with other known mucoadhesives (chitosan and poly(d,l-lactic acid)) in addition to WGA. The results are shown in Figures S4–S7. As can be seen from the results, the performance of the peptides is comparable to or slightly better than that of the known mucoadhesives, suggesting that the peptides can adhere to the site of interest and could confer a targeting benefit over longer periods of time.

### Binding Kinetics Measurement and Dissociation Constant Calculation for Peptide Binding to Mucins Under Physiological Conditions

The mucin-binding kinetic constants and dissociation constant *k*_d_ measured via biolayer interferometry (BLI) for the identified hits under physiological conditions are found in Table S1. The target proteins (MUC2 and MUC5AC) were suspended in PBS buffer, in which the pH was adjusted to that of the organs of interest (pH 7.2 for MUC2 and pH 1.82 for MUC5AC). The *k*_d_ values for the binding of M2.3 and M2.4 to MUC2 (on-target binding) at pH 7.2 were 7.11 × 10^–9^ M and 1.12 × 10^–7^ M, respectively, while the *k*_d_ values for the binding of M5.2 and M5.3 to MUC5AC (on-target binding) at pH 1.82 were 7.88 × 10^–9^ M and 1.54 × 10^–9^ M, respectively. This nanomolar to micromolar affinity is comparable to that reported for cyclic peptides binding to other targets.^43,44^ For binding to MUC2, we selected M2.3 for further testing, as its *k*_d_ value for MUC2 binding was 1–2 orders of magnitude greater than for MUC5AC binding (where the *k*_d_ value was 6.54 × 10^–8^ M). M5.2 was identified as the MUC5AC-targeting candidate, with its *k*_d_ value for MUC5AC binding being 2 orders of magnitude stronger than for MUC2 binding (where the *k*_d_ value was 1.12 × 10^–7^ M). The identified binding (nanomolar affinity) supports the selection of M2.3 and M5.2 and narrows the four peptide hits with high selectivity from tissue screening. Binding curves for the peptides to the mucins (both on-target and off-target) at various physiologically relevant pH values are shown in Figure S3. These values for on-target binding affinity are comparable to or slightly stronger than those measured for other previously identified site-selective glycan-targeting molecules, such as *N*-glycan-targeting peptides^45^ and sialoglycan-targeting glycan recombinant antibody binders.^46^

Dissociation constants can be calculated in numerous ways, so examining the relative magnitudes of the adsorption and desorption rate reactions can provide additional insight into the relative importance of each reaction and, thus, its potential use in the body. A faster rate of adsorption indicates faster binding to the target of interest, while a slower rate of desorption indicates that the peptide-target bond is retained for a longer period. For the purposes of designing a location-specific ligand, adsorption rates should be faster for on-target binding than for off-target binding (to show selectivity), while desorption rates should ideally be as slow as possible to promote retention of the peptide/mucin bond. Specifically, looking at the *k*_off_ values for all of the peptide sequences (which indicate the rate of desorption of the peptides from the mucin), the binding shows consistent dissociation rates on the order of magnitude of 10^–3^ s^–1^, demonstrating that the rate of desorption was relatively constant across peptides, mucins, and different pH environments. Conversely, the *k*_on_ values, which indicate the rate of binding of the peptides to mucins, varied significantly. For M2.3 and M2.4, the *k*_on_ values for on-target binding were 6.99 × 10^5^ M^–1^s^–1^ and 5.77 × 10^4^ M^–1^ s^–1^, respectively, while the *k*_on_ values for off-target binding were 2.70 × 10^4^ M^–1^s^–1^ and 2.95 × 10^4^ M^–1^s^–1^, respectively. For M5.2 and M5.3, the *k*_on_ values for on-target binding were 4.03 × 10^5^ M^–1^s^–1^ and 1.01 × 10^6^ M^–1^s^–1^, respectively, while the *k*_on_ values for off-target binding were 3.99 × 10^4^ M^–1^s^–1^ and 6.33 × 10^4^ M^–1^s^–1^, respectively. Larger magnitudes of *k*_on_ values suggest faster binding, while smaller magnitudes of *k*_off_ values suggest slower desorption and a longer retention time. The strongest peptide candidates (M2.3 for MUC2, and M5.2 and M5.3 for MUC5AC) exhibited faster binding rates of reaction (greater than 10^5^ M^–1^s^–1^) compared to their binding to the off-target mucins (∼10^4^ M^–1^s^–1^).

### *In Vivo* Gastrointestinal Localization of Peptides

**Figure 4:**
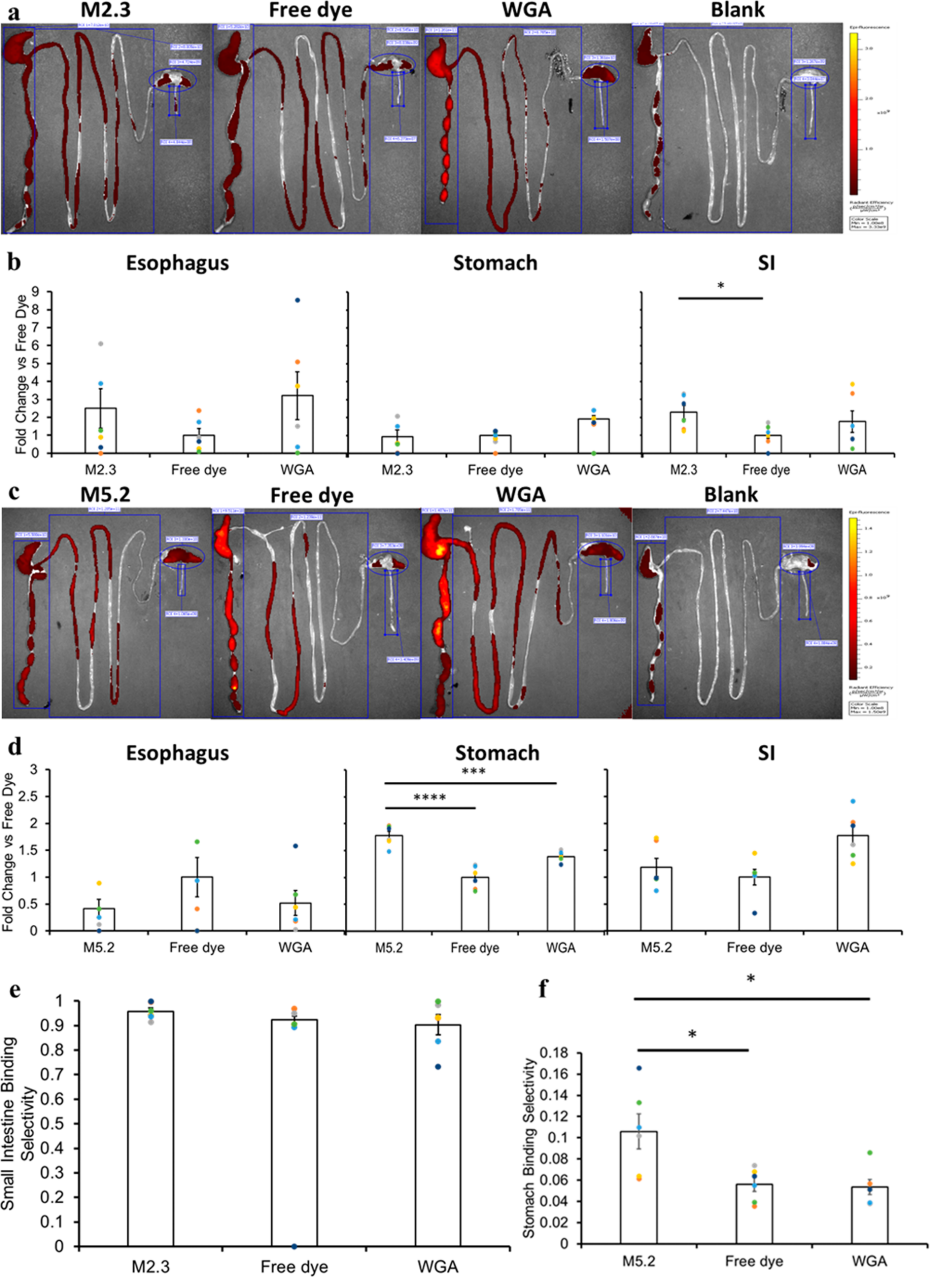
*In vivo* analysis of top predicted peptide hits. Representative IVIS images are shown for M2.3, along with the free AF647 and WGA-AF647 controls and blank PBS (a). The fluorescence intensity in each organ (esophagus, stomach, and small intestine) is shown for M2.3 and the controls (b); the results shown subtracted the tissue background and the fluorescence values from the blank group rats. Similar IVIS images (c) and tissue fluorescence results (d) are also shown for M5.2, free AF647, and WGA-AF647. Finally, the overall selectivity is shown for M2.3 in (e) and M5.2 in (f), each compared to the controls. Upper values in fluorescence scale bars in (a) and (c) are defined as follows: (a) 3 × 10^9^, (c) 1.2 × 10^9^. Statistical significance is designated by *p < 0.05, ***p < 0.005, ****p < 0.001 (two-tailed Student’s *t*-test for single comparisons, one-way ANOVA and post-hoc Bonferroni for multiple comparisons). *n* = 6 for *in vivo* experiments.

To measure the gastrointestinal localization of these peptides *in vivo,* peptides M2.3 and M5.2 with conjugated AF647 dye (as a model drug) were administered via oral gavage to fasted Sprague–Dawley rats, along with unconjugated AF647 dye and WGA-AF647. After 6 h, localization was measured by using fluorescent imaging on the rats’ GI tracts. The major results of the total binding fluorescence and organ-specific binding fluorescence for M2.3, as well as the total binding fluorescence and organ-specific binding fluorescence for M5.2, along with results for the AF647 and WGA-AF647 in each case, are shown in Figure 4, with more detailed results found in Figure S9. All results have been adjusted by subtracting the fluorescence from tissue from untreated rats. Representative IVIS images are shown in Figure 4a and c, while analyses of the results are shown in Figure 4b,d −f. Graphical results from the IVIS images in Figure 4a are shown in Figure 4b and e, while graphical results from the IVIS images in Figure 4c are shown in Figure 4d and f.

In general, the hits showed a modest increase in total binding throughout the GI tract when compared to the free dye (Figure 4b). M2.3 showed the greatest binding in the small intestine, with a 2-fold improvement over the free dye (Figures 4band S9d). While selectivity results were slightly affected by the normal passage of particles through the GI tract, M2.3 showed higher selectivity for the small intestine than the free dye and comparable selectivity to the WGA-dye conjugate (Figure 4e). It showed a significant improvement over the free dye, while the WGA-dye conjugate (which also showed some improvement) did not demonstrate a significant improvement over the free dye. M5.2 showed the greatest magnitude of stomach binding when compared to the controls, with a 2-fold improvement compared to the free dye and a 1.29-fold improvement compared to the WGA-dye conjugate (Figures 4dand S9f). Additionally, M5.2 showed a 2-fold improvement in stomach-binding selectivity compared with the WGA-dye conjugate (Figure 4f), demonstrating that M5.2 can improve localization to the stomach compared to a general mucoadhesive. Because the 6 h time point is longer than the general GI transit time in fasted rats,^47^ the greater amounts of fluorescence from the peptides (compared to the free dye and WGA-dye conjugate) in the regions of interest indicate that retention could be improved in these regions. While the improvements shown here are relatively modest (particularly in comparison to the known mucoadhesive WGA), they represent an important starting point in utilizing the differences in mucin expression to aid in targeting different regions of the GI tract.

## Conclusions

Oral drug delivery is a crucial method of drug administration due to its minimal invasiveness compared to other forms, such as intravenous or intramuscular drug delivery. However, one major challenge is the ability to localize a sufficient quantity of the drug at the necessary site of absorption in order to reach clinically relevant concentrations.^48^ To address this limitation, we identified and validated short-chain cyclic peptides that could be used to bind strongly and selectively to mucin glycoproteins present in the mucus layers of the GI tract. These layers cover the epithelial tissue in the GI tract, facilitate nutrient diffusion into the epithelium, and protect the surface from pathogenic attack.^49^ Mucin glycoproteins are synthesized in goblet cells throughout the GI epithelium and are secreted to form the mucus layers.^50^

Since these mucins are only expressed in specific geographic areas within the GI tract, we hypothesized that identifying peptides that could serve as targeting ligands would enable the development of location-specific mucoadhesive systems. These systems could bind strongly and selectively to certain regions of the GI tract, allowing for superior localization and retention, thereby improving on-target delivery efficacy. We selected a cyclic peptide ligand approach to increase the ligands’ stability within the proteolytic environment of the GI tract (particularly the stomach), as cyclic peptides have been shown to better resist the actions of proteases and other digestive enzymes.^30^ The enhanced proteolytic stability conferred by cyclic peptides is observed both in the stomach^51^ and small intestine,^52^ with potential benefits due to the inhibition of protease activity compared to linear peptides^53^ and the inhibition of degradation by microflora present in the GI tract.^54^ Because the targeting capability of any ligand is dependent on its structural retention upon exposure to the gastrointestinal environment *in vivo*, utilizing a cyclic peptide with enhanced stability should theoretically improve its targeting capability compared to using a linear peptide.

To develop this method of targeting, we first identified the peptides of interest by employing phage display against the MUC2 and MUC5AC targets. We did this by using a phage library containing 1.28 × 10^9^ unique cyclic peptide sequences (or every combination of seven amino acids flanked on either side by two cysteine residues to create a “loop” structure). Through iterative cycles of “panning” and “amplification,” the large library size was efficiently enriched to a set of eight unique peptide sequences that exhibited selective binding to the two mucin targets. After further validation of the phage hits using phage ELISA, the four top-performing hits were selected for further validation. These hits were then synthesized, and their mucin-binding performance was investigated through experimentation on porcine tissue; the top hits from these studies were then studied in rats. The binding kinetics were observed for the top hits, revealing high-affinity (micromolar to nanomolar) and selective (reduction by an order of magnitude for off-target interactions) binding, primarily driven by faster binding to the mucin. Subsequently, the top hits were orally administered to a rat model *in vivo* (conjugated to a fluorophore), demonstrating greater localization and possibly enhanced retention of the bound fluorophore compared with the controls; this could potentially be applied to other bound cargos such as drugs or drug-loaded particles. The results indicate that the most promising identified peptide hits significantly enhance the localization of bound cargo at a specific site (in this case, the stomach or small intestine) within the GI tract upon oral administration.

The *in vivo* results showed a 2-fold increase in intestinal binding of a fluorophore conjugated to peptide M2.3 when compared to the unconjugated fluorophore, and a 2-fold increase in stomach binding of a fluorophore conjugated to peptide M5.2 compared to the unconjugated fluorophore (with a 1.29-fold increase compared to a wheat germ agglutinin-conjugated fluorophore) after 6 h. Greater binding to the stomach and intestine can allow a reduced dosage of the drug to be administered with the same efficacy.

While our results demonstrate that this approach has promise in enabling site-specific targeting of peptide-bound cargos, we recognize a few challenges. One consideration is that the identified peptides are not optimized for the *in vivo* targeting of the mucin layers. To address this, methods, such as *in vivo* phage display, could be employed to preferentially select peptides that target well *in vivo.* Another consideration is that our method of measuring the localization of the peptides *in vivo* could be affected by factors other than the composition of the lectin. For example, the normal passage of material through the GI tract would result in greater accumulation in the small intestine over time (before excretion from the body), and that might influence the apparent binding and selectivity of peptides and controls to MUC2 in the small intestine. To address this, imaging of live animals (such as mice) could be performed more often to establish a more robust set of data over different time periods. Both of these approaches could be used to improve the *in vitro–in vivo* translation of candidate peptides.

When looking at the amino acid motifs present in some of the top hits, we see some similarities to known mucus-binding proteins in nature. For example, the motif “ASS” identified in multiple candidate peptides for MUC2 and MUC5AC binding, is found in the internalin E protein found in *Listeria monocytogenes*, a known mucus-binding pathogen which is found throughout the human GI tract.^55^ Internalin E is part of a class of proteins that facilitate *L. monocytogenes* intestinal invasion.^56^ Likewise, the motif “TAL” (a MUC5AC-specific motif) is found in the amino acid sequence of 32-Mmubp, a mucus-binding protein found on the cell surface of *Lactobacillus fermentum* BCS87,^57^ a bacterial species found in the human stomach.^58^ The capacity of our peptide library to identify these motifs suggests that our selection method can identify successful mucin-binding peptide motifs.

These peptides could enable various gastrointestinal applications. First, this targeting can provide a more effective treatment of local GI diseases by ensuring that the drug is mostly released at the site of the disease. For example, this could aid in the treatment of stomach ailments caused by *H. pylori* infection, as most of the antibiotic could be delivered to the stomach rather than losing some of the drug to release and absorption in the small intestine. This could potentially lead to the development of shorter-term drug regimens while maintaining treatment efficacy, thereby reducing the risk of antibiotic resistance.^59^ Furthermore, providing location-specific mucoadhesion can prolong residence within the GI tract and enable selective drug release at specific GI tract sites, which can facilitate the absorption of certain drugs. For example, furosemide is preferentially absorbed in the stomach, with research by Klausner et al. demonstrating that developing a gastroretentive formulation can triple the length of the absorption phase of furosemide in the stomach.^60^ The system developed in this work shows similar sequestration of a payload and can potentially be applied in a versatile manner to drugs such as furosemide. Conversely, some drugs exhibit poor stomach absorption in the stomach but higher bioavailability when absorbed in the small intestine or colon. For example, Sampathi et al. demonstrated that preparing curcumin nanosuspensions for colonic delivery can improve bioavailability 4-fold.^61^

Further improvements can enhance the conclusions drawn from this work. First, screening gastrointestinal targets with increasing specificity can refine the selectivity of the approach. Our method utilized differences in mucin composition to achieve location-specific drug delivery to various organs within the GI tract (in this study, the stomach and small intestine). We could potentially provide a strategy to localize drug action. However, targeting specificity can be further enhanced by identifying more specific targets within these regions that exhibit different expressions. For example, the intestinal epithelium contains a variety of different cell types (such as enterocytes, goblet cells, Paneth cells, Peyer’s patches, dendritic cells, proliferating stem cells, and neuroendocrine cells), each with its own unique functions.^62^ Developing methods to target these specific cell types can enhance the ability to modulate their functions selectively without affecting neighboring cell types.^63^ Additionally, the glycosylation of the mucins can be altered in various disease states,^64^ and targeting molecules that recognize these glycosylation changes can improve targeting toward diseased mucosa. This approach can enhance drug delivery to the disease site, thereby increasing efficacy and reducing side effects associated with off-target release. Our approach of using mucins as a target (which have a relatively broad spatial range, even if they are selectively expressed in certain locations) can be seen as the first step in utilizing the complex biology of the GI tract to aid in targeted drug delivery.

Furthermore, the selected peptides can be engineered by altering their composition to explore alternative sequences or structures that exhibit both robust performance and resistance to degradation in the GI tract. For instance, the strategic design of peptide sequences to maximize the presence of helpful motifs (such as the “ASS” motif for MUC2-selective binders or the “TAL” motif for MUC5AC-selective binders) can enhance the binding strength to mucins. Moreover, exploring unconventional peptide structures, such as nonconventional amino acids, to create α/β peptides^65^ or retro-inverso peptides^66^ can augment structural diversity and resist proteolysis in the GI tract.

The approach outlined in this paper and the mucin-selective peptides identified here have potential applications in the field of nanotechnology. First of all, the peptides can be conjugated to the surface of functionalized nanoparticles using conjugation chemistries such as click chemistry^67^ and pyridyl disulfide chemistry.^68^ This approach would allow the peptides to serve as targeting “ligands” that enable site-specific or systemic oral delivery of therapeutic agents, as described above. Second, the same type of approach (conjugation of peptides to fluorescent nanoparticles) can be used to target diagnostics or imaging agents to specific sites in the GI tract.^69^ Finally, the approach taken to use the biological differences present in the GI tract as a mechanism for site-specific targeting can be utilized for other bionanotechnology applications, such as cancer targeting^70^ and *in vivo* bioanalysis,^71^ among others.

In conclusion, we have identified and validated cyclic peptide-based targeting ligands that exhibit strong and selective binding to tissue-specific mucin glycoproteins (MUC2 and MUC5AC) at the *in vitro*, *ex vivo*, and *in vivo* levels. From this, we have demonstrated that these peptides can be used to localize and retain bound cargo *in vivo*. Given their potential compatibility with various drug carriers, which can themselves contain a variety of orally administered drugs, these peptides could possibly aid in creating a versatile drug delivery platform suitable for diverse applications.

## Methods and Experimental

### Materials

The Ph.D. C7C bacteriophage library was purchased from New England Biolabs. Tetracycline, Tween-20, and Blue-White Select Screening Reagent were purchased from Sigma-Aldrich. 20× Tris-buffered saline (TBS) was purchased from Invitrogen. Luria Bertani powder media and Bacto-Agar were purchased from Fisher Scientific. Poly(ethylene glycol)-8000 and sodium chloride were purchased from VWR. The CCCTCATAGTTAGCGTAACG DNA primer was purchased from Integrated DNA Technologies. Anti-M13 horseradish peroxidase (HRP) mouse monoclonal antibody was purchased from Abcam. The 3,3′,5,5′-tetramethylbenzidine (TMB) substrate reagent set was purchased from Fisher Scientific. Cy5-conjugated cyclic peptides were synthesized and purchased from the Koch Institute Swanson Biotechnology Center, Biopolymers and Proteomics Core Facility. Alexa Fluor 647-Maleimide and Alexa Fluor 647-conjugated wheat germ agglutinin were purchased from Thermo Fisher Scientific. Cy5-chitosan was purchased from NanoCS. Cy5-poly(d,l-lactic acid) was purchased from CD Biosciences. Porcine tissue was procured from local abattoirs and laboratory-maintained pigs.

### *In Vitro* Phage Display

For each mucin of interest, 5 μg of mucin protein was diluted in 150 μL of Tris-buffered saline (TBS) and added to 2 wells of a microtiter plate. The plate was left overnight in a humidified, sealed container with agitation at 50 rpm. After the overnight mucin coating, the solution was poured out, and 300 μL of blocking buffer was added to the wells for 1 h. After the 1 h waiting period, the blocking buffer was poured out, and the wells were washed three times with 300 μL of Tris-buffered saline with TBST (TBS + 0.1% (v/v) Tween-20) to remove nonspecific binding on the mucin layer coating. Next, 5 μL of the C7C library diluted with 95 μL of TBST was added to each blocked well and placed on shaker for 45 min, before pouring out the nonbinding phage and washing the plate ten times with TBST. A 0.2 M glycine-HCl elution buffer (pH 2.2, 100 μL) was added to each well and placed on a shaker for 20 min to release the mucin-layer binding peptides on the plate as unamplified eluate, with the pH neutralized by 30 μL of neutralization buffer (1 M Tris-HCl, pH 9).

*E. coli* liquid cultures were prepared by adding *E. coli* cells to 20 mL of LB media and placing them in shaking conditions at 37 °C and 250 rpm for 2 h and 45 min. The unamplified eluate (1 μL of which was saved for titering) was added to the *E. coli* cultures under the same conditions for 4 h and 45 min for amplification. After amplification, the cultures were centrifuged for 10 min at 12,000 *g* RCF at 4 °C, and the supernatant was collected and spun again under the same conditions for 5 min. The top 16 mL of supernatant was collected and mixed with 4 mL of PEG/NaCl (20% (w/v) PEG-8000/2.5 M NaCl in water) to promote precipitation of the mucin-binding peptide-containing phage and left overnight.

Titering was performed to measure the amount of phages present in the solution. From the obtained unamplified eluate, 1 μL of unamplified eluate was added to 999 μL of LB solution to obtain a 10³ dilution. Three 200 μL *E. coli* liquid cultures were prepared for eluate sample mixtures: 10 μL of diluted unamplified eluate, 10 μL of diluted amplified eluate, and one culture with no eluate (no phage). Top agar solutions (7 g/L Bacto-Agar in LB) were melted and cooled for the *E. coli* + eluate phage solutions to be poured onto three prepared indicator-containing agarose plates (0.1% (v/v) Blue-White Select Screening Reagent in 15 g/L Bacto-Agar in LB medium solution, 10 mL total per plate) and left overnight. The indicator solution used for the agarose plates was obtained from MilliporeSigma. Successful amplification was indicated by titering plates that presented fully blue amplified eluate plates and unamplified eluate plates with distinct phage plaques.

Following successful phage amplification, the *E. coli* culture + PEG/NaCl phage solution was centrifuged for 15 min at 12,000 *g* RCF at 4 °C, and the supernatant was discarded. The remaining pellet was dissolved in 1 mL of TBS, and the phage was reprecipitated with 200 μL of PEG/NaCl, incubating for 60 min on ice. After the incubation period, the sample was spun for 12 min at 14,000 rpm at 4 °C, and the supernatant was removed without disturbing the pellet. The pellet was resuspended in 200 μL of TBS to produce the purified amplified eluate sample. Dilutions of 10^7^ and 10^9^ of the amplified eluate samples in LB were titered to confirm successful amplification.

Two more rounds of panning and amplification were performed on the amplified eluate sample following the same procedure as the first round, except that TBS + 0.5% Tween-20 was used for washing conditions rather than TBST. The phage titering of the first-round amplified eluate samples was used to determine the volume of the sample needed to achieve a total phage count of ∼10^11^ virions. For the third round of panning, the unamplified eluate obtained was diluted to 10^2^ and 10^4^ for titering.

After finishing the third round of panning, 20 blue plaques were individually collected and amplified in 1 mL of LB (with 1% overnight *E. coli* culture). Ten μL of each of the 20 prepared overnight plaque samples were dispensed into individual culture tubes (20 tubes in total), and each tube was diluted with 990 μL of LB solution. The tubes were incubated and mixed for 4 h and 45 min at 250 rpm at 37 °C. After incubation, the samples were spun for 10 min at 14,000 rpm at 4 °C, and the top 900 μL of the supernatant from each sample was collected as the amplified phage stock for each plaque.

The phage samples were prepared and sent to GENEWIZ for DNA sequencing. For the preparation, 6 μL of DNA primer (CCCTCATAGTTAGCGTAACG) was diluted in 114 μL of IDTE (10 mM Tris, 1 mM ethylenediaminetetraacetic acid (EDTA), pH 8) buffer solution (obtained from Integrated DNA Technologies (IDT)). For the phage samples, 50 μL of each amplified phage stock was sent to GENEWIZ.

### Phage ELISA

To validate the binding performance of the individual phage clones and to demonstrate their binding performance to the identified targets, we performed an enzyme-linked immunosorbent assay (ELISA). First, mucin was coated onto the surface of a plastic plate overnight using the same process as that performed for the original phage display. The next morning, three wells of each phage clone were prepared, with each phage solution having a titer of 10^7^ PFU/μL to ensure a fair comparison between the clones. In addition, three negative controls—a dilution of the original library, a sample of wild-type phage without a peptide insert, and a sample without any phage—were prepared similarly. After the mucin-containing plate was blocked and washed, the samples were added to the mucin-bound wells, and a typical panning experiment took place.

However, instead of eluting the bound phage, 100 μL of 1:2000 anti-M13/HRP mouse monoclonal antibody was added to each well, and the antibody was allowed to incubate with the wells for 30 min. During this time, the antibody binds to the phage present in the wells. After the plates were washed to remove any unbound antibody, 100 μL of TMB substrate was added to each well and allowed to incubate for 20 min, covered with foil. This produced a colorimetric change from clear to blue as the TMB reacted with the HRP, which was quenched by adding 50 μL of 2 N H2SO4 to each well, which then turned the solutions yellow. The resulting solutions were analyzed by absorbance spectroscopy at a wavelength of 450 nm to determine the relative amount of phage present in each well.

### *Ex Vivo* Fluorescent Screening of Top Peptide Ligands to Porcine Tissue

The top-performing hits from phage display were tested on *ex vivo* segments of porcine mucin-containing tissues (jejunal, stomach, and esophageal tissues) obtained from local abattoirs or laboratory-maintained pigs. Tissue was used either immediately after acquisition or after storage at −80 °C; tissue was used at most 1 week after acquisition and storage at −80 °C. After acquisition and equilibration to room temperature, the tissue was washed twice with 25 mL (each) of cold 1× PBS to remove undigested food and other debris. After washing and cleaning, the tissue was mounted between two magnetic plates with holes corresponding to a 48-well plate, as previously described,^72^ such that the luminal side of the tissue was facing up (Figure S1). After the tissue was mounted, the amount of peptide/protein corresponding to 40 μg of AF647 was suspended in 200 μL of 1× PBS and added to the wells created by the magnetic plates. This was done 3× for each formulation to perform the experiment in triplicate. In addition, plain 1× PBS was added to one well to serve as the background measurement. After the peptides were added, the plate setup was covered with aluminum foil and allowed to incubate for 1 h at room temperature with gentle agitation (50 rpm). After incubation, the nonbinding peptides were removed by pouring out the solutions onto a clean paper towel, and the setup was washed three times by adding 200 μL of 1× PBS to each well and gently agitating the setup for 30 s before removing the washing solution. After washing was complete, fluorescence imaging was performed using an IVIS Spectrum fluorescent imaging system (PerkinElmer), with an automatically calculated exposure time (usually ∼1 s), a 20 cm × 20 cm field of view, and an excitation/emission of 640/680 nm, respectively. The imaging results were analyzed using Living Image software to calculate the total fluorescence intensity for each well, and the results for each hit or control were averaged across different wells. The negative control used was unconjugated AF647, while the positive control was WGA-AF647, since WGA is a known lectin that binds glycoproteins.

To better account for tissue-to-tissue variability, the results for each trial were reported as scores rather than using the raw fluorescence intensity. For each trial, the calculated fluorescence intensities were scaled between the negative and positive controls as follows:
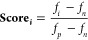


Here, score_*i*_ represents the hit score for hit *i* (that is reported), *f*_*i*_ represents the mean fluorescence intensity for hit *i*, and *f*_*n*_ and *f*_*p*_ represent the mean fluorescence intensities for free AF647 and WGA-AF647, respectively. As such, the score for the negative control will always be 0, and the score for the positive control will always be 1, while the score for the “hits” will be closer to (or greater than) 1 for successful hits and closer to 0 for unsuccessful hits.

The selectivity of a hit *i* to a particular mucin *m* is calculated as follows:
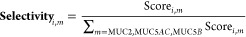


Here, the scores shown represent the mean hit score across multiple trials (*n* = 3) for a particular mucin-containing tissue.

### Measurement of *In Vitro* Binding Kinetics for Top Peptide Ligands

Binding kinetics for the successful mucin-binding hits were measured using biolayer interferometry (BLI).^73^ In BLI, a protein target is immobilized on the surface of a biosensor, creating a thin optical layer. These coated sensors are then exposed to a solution of the ligand (in this case, the small molecule and peptide hits), allowing the ligand to associate and dissociate with the protein target. This association/dissociation behavior changes the thickness of the optical layer, and the instrument correlates this change in thickness with binding and desorption. By testing different concentrations of the ligand, the kinetic rate constants and dissociation constant (*k*_d_) can be measured by fitting the resulting binding curves according to a binding model.^73^

For the analysis of mucin-binding kinetics, mucins MUC2 and MUC5AC were used as the protein targets, and the “hits” were used as the ligands of interest. Mucins were biotinylated and suspended in buffers with different pH levels (MUC2 in PBS with pH 7.2 and PBS-HCl with pH 3.35, MUC5AC in PBS-HCl with pH 1.82) that mimic the pH of the normal small intestine,^74^ the small intestine during inflammatory bowel disease,^75^ and the normal stomach,^76^ respectively. Hits were dissolved in the same buffer to minimize the optical differences among the buffer, mucin solution, and analyte solution. BLI was carried out on an Octet RED96 platform in five steps. First, a baseline step of 60 s was carried out. Then, biotinylated mucins were immobilized onto the surface of a streptavidin sensor during a loading step of 300 s. Next, the loaded tips were allowed to re-equilibrate in another baseline step for 60 s. Following that, the loaded tips were allowed to incubate in a solution of analyte (an association step) for 300 s; this allowed the hits to interact with the mucin, changing the thickness of the optical layer and generating a response from the optical measurement. Finally, the tips were moved to buffer wells, and the hits were allowed to dissociate from the mucin for 300 s. Data were analyzed, and ligand-only and hit-only controls were used as references to properly characterize the binding performance of the hits.

Binding of the hits to the mucins generally follows one of two different mechanisms. The first is a 1:1 bimolecular model in which the molecule binds to a specific spot on the mucin. The reaction can, therefore, be described using the following model:^77^
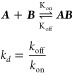


In this model, *k*_on_ represents the rate of association of the hit to the mucin, while *k*_off_ represents the rate of dissociation of the molecule from the molecule-mucin complex. The dissociation constant is calculated as the ratio of these two rate constants.

The second model is a 2:1 heterogeneous model, in which the molecule binds at two locations on the mucin (each with its own kinetic rate constants and dissociation constant).^78^ These reactions are described using the following model:
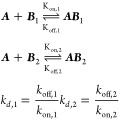


The rate constants for each interaction are equivalent to those of the single interaction present in the 1:1 model, while each interaction has its own dissociation constant. This was calculated similarly to the 1:1 model.

### *In Vivo* Gastrointestinal Localization of Top Peptide Ligands

All animal studies were carried out under the supervision of MIT’s Division of Comparative Medicine and in compliance with NIH’s *Principles of Laboratory Animal Care*, following the guidelines set forth by MIT’s Committee on Animal Care. Sprague–Dawley rats (200–300 g, a mix of male and female) were obtained from Charles River Laboratories and allowed to acclimate to their new conditions for 1 week, following which their diet was adjusted to an alfalfa-free diet (AIN-93M, TestDiet) for at least 7 days prior to the study in order to reduce background fluorescence. The night before the study, the diet was removed, and the rats were fasted; they received no food for the duration of the experiment, but were provided normal access to regular water. On the day of the study, rats (*n* = 6) were randomly allocated to different groups and administered formulations (hits, free dye, WGA-AF647, blank) via oral gavage. For rats dosed with the hits and controls, peptides and controls were administered at an amount of peptide/protein corresponding to 200 μg of AF647 in 400 μL of 1× PBS (1 mg/kg), while the blank was administered 400 μL of PBS only. After 6 h, the rats were euthanized through CO_2_ exposure and asphyxiation. The GI tract was extracted intact and placed in 50 mL of 1× PBS to remove excess blood from the outside of the GI tract. Fluorescence imaging was performed on the extracted GI tracts using the same IVIS system as before, with an automatically calculated exposure time (ranging between 1 and 15 s depending on fluorescent intensity), a 23 cm × 23 cm field of view, and excitation/emission of 570/620 nm, respectively. Imaging results were obtained for the esophagus, stomach, small intestine, and colon, and these were analyzed by using the Living Image software. The fluorescence values for the hits and controls were defined as the total fluorescence intensity for the segment of tissue with the average fluorescence value of the blank for that segment subtracted (to reduce the effect of background autofluorescence), while the selectivity for that segment was calculated as the fluorescence value divided by the sum of the fluorescence values across all three segments of tissue (esophagus, stomach, and intestine).

### Statistical Analysis

To determine whether differences between two groups were statistically significant, a two-tailed Student’s *t*-test was used. One-way analysis of variance (ANOVA) and post-hoc Bonferroni tests were used for multiple comparisons. A *p*-value of less than 0.05 was considered to be statistically significant.

## Supporting Information Available

Specific mucin-binding kinetic information for top hits (kinetic constants and binding curves at different pH) (Table S1); raw fluorescence counts and nanoparticle “score” for each ex vivo experiment (Tables S2–S7); these tables show the fluorescence counts for each fluorescently tagged peptide, as well as the controls, binding to on-target and off-target mucin-containing tissues, as well as the calculated nanoparticle “scores” as defined in the Methods/Experimental section; a schematic of *ex vivo* tissue binding setup (Figure S1);fluorescence comparison between experimental groups (Figure S2); tissue-by-tissue ex vivo binding results (Figure S3); time-lapse ex vivo binding study results (Figures S4–S7); mucin-binding kinetic curves (Figure S8);In vivo binding distribution results (Figure S9). (PDF)

## References

[ref1] Homayun B.; Lin X.; Choi H.-J. Challenges and Recent Progress in Oral Drug Delivery Systems for Biopharmaceuticals. Pharmaceutics 2019, 11 (3), 129 10.3390/pharmaceutics11030129. 30893852 PMC6471246

[ref2] Polonsky W. H.; Henry R. R. Poor medication adherence in type 2 diabetes: Recognizing the scope of the problem and its key contributors. Patient Preference Adherence 2016, 10, 1299–1307. 10.2147/PPA.S106821. 27524885 PMC4966497

[ref3] Loretz B.; Foger F.; Werle M.; Bernkop-Schnurch A. Oral gene delivery: Strategies to improve stability of pDNA towards intestinal digestion. J. Drug Target 2006, 14 (5), 311–319. 10.1080/10611860600823766. 16882551

[ref4] aLehr C.-M.; Poelma F. G. J.; Junginger H. E.; Tukker J. J. An estimate of turnover time of intestinal mucus gel layer in the rat in situ loop. Int. J. Pharm. 1991, 70 (3), 235–240. 10.1016/0378-5173(91)90287-X.

[ref5] Chen G.; Kang W.; Li W.; Chen S.; Gao Y. Oral delivery of protein and peptide drugs: From non-specific formulation approaches to intestinal cell targeting strategies. Theranostics 2022, 12 (3), 1419–1439. 10.7150/thno.61747. 35154498 PMC8771547

[ref6] Lambkin I.; Pinilla C. Targeting approaches to oral drug delivery. Expert Opin. Biol. Ther. 2002, 2 (1), 67–73. 10.1517/14712598.2.1.67. 11772341

[ref7] Alai M.; Lin W. J. Application of nanoparticles for oral delivery of acid-labile lansoprazole in the treatment of gastric ulcer: *In vitro* and *in vivo* evaluations. Int. J. Nanomed. 2015, 10 (null), 4029–4041. 10.2147/IJN.S82366. PMC4476456 26124659

[ref8] Friend D. R. New oral delivery systems for treatment of inflammatory bowel disease. Adv. Drug Delivery Rev. 2005, 57 (2), 247–265. 10.1016/j.addr.2004.08.011. 15555741

[ref9] Krishnaiah Y. S. R.; Khan M. A. Strategies of targeting oral drug delivery systems to the colon and their potential use for the treatment of colorectal cancer. Pharm. Dev. Technol. 2012, 17 (5), 521–540. 10.3109/10837450.2012.696268. 22681390

[ref10] Feldman M.; Cryer B. Aspirin absorption rates and platelet inhibition times with 325-mg buffered aspirin tablets (chewed or swallowed intact) and with buffered aspirin solution. Am. J. Cardiol. 1999, 84 (4), 404–409. 10.1016/S0002-9149(99)00324-0. 10468077

[ref11] Pinto J. F. Site-specific drug delivery systems within the gastro-intestinal tract: From the mouth to the colon. Int. J. Pharm. 2010, 395 (1), 44–52. 10.1016/j.ijpharm.2010.05.003. 20546856

[ref12] Guo F.; Zhang M.; Gao Y.; Zhu S.; Chen S.; Liu W.; Zhong H.; Liu J. Modified nanoparticles with cell-penetrating peptide and amphipathic chitosan derivative for enhanced oral colon absorption of insulin: Preparation and evaluation. Drug Delivery 2016, 23 (6), 2003–2014. 10.3109/10717544.2015.1048489. 26181840

[ref13] Hua S.; Marks E.; Schneider J. J.; Keely S. Advances in oral nano-delivery systems for colon targeted drug delivery in inflammatory bowel disease: Selective targeting to diseased versus healthy tissue. Nanomedicine 2015, 11 (5), 1117–1132. 10.1016/j.nano.2015.02.018. 25784453

[ref14] Streubel A.; Siepmann J.; Bodmeier R. Gastroretentive drug delivery systems. Expert Opin. Drug Delivery 2006, 3 (2), 217–233. 10.1517/17425247.3.2.217. 16506949

[ref15] Subramanian D. A.; Langer R.; Traverso G. Mucus interaction to improve gastrointestinal retention and pharmacokinetics of orally administered nano-drug delivery systems. J. Nanobiotechnol. 2022, 20 (1), 362 10.1186/s12951-022-01539-x. PMC9356434 35933341

[ref16] ades Rieux A.; Fievez V.; Garinot M.; Schneider Y. J.; Preat V. Nanoparticles as potential oral delivery systems of proteins and vaccines: A mechanistic approach. J. Controlled Release 2006, 116 (1), 1–27. 10.1016/j.jconrel.2006.08.013. 17050027

[ref17] Smart J. D. The basics and underlying mechanisms of mucoadhesion. Adv. Drug Delivery Rev. 2005, 57 (11), 1556–1568. 10.1016/j.addr.2005.07.001. 16198441

[ref18] Peppas N. A.; Sahlin J. J. Hydrogels as mucoadhesive and bioadhesive materials: A review. Biomaterials 1996, 17 (16), 1553–1561. 10.1016/0142-9612(95)00307-X. 8842358

[ref19] aBravo-Osuna I.; Vauthier C.; Farabollini A.; Palmieri G. F.; Ponchel G. Mucoadhesion mechanism of chitosan and thiolated chitosan-poly(isobutyl cyanoacrylate) core-shell nanoparticles. Biomaterials 2007, 28 (13), 2233–2243. 10.1016/j.biomaterials.2007.01.005. 17261330

[ref20] Derjaguin B. V.; Toporov V.; Muller V. M.; Aleinikova I. N. On the relationship between the electrostatic and the molecular component of the adhesion of elastic particles to a solid surface. J. Colloid Interface Sci. 1977, 58 (3), 528–533. 10.1016/0021-9797(77)90162-X.

[ref21] Yun Y.; Cho Y. W.; Park K. Nanoparticles for oral delivery: Targeted nanoparticles with peptidic ligands for oral protein delivery. Adv. Drug Delivery Rev. 2013, 65 (6), 822–832. 10.1016/j.addr.2012.10.007. PMC3574626 23123292

[ref22] Jin Y.; Song Y.; Zhu X.; Zhou D.; Chen C.; Zhang Z.; Huang Y. Goblet cell-targeting nanoparticles for oral insulin delivery and the influence of mucus on insulin transport. Biomaterials 2012, 33 (5), 1573–1582. 10.1016/j.biomaterials.2011.10.075. 22093292

[ref23] Pasqualini R.; Koivunen E.; Ruoslahti E. αv Integrins as receptors for tumor targeting by circulating ligands. Nat. Biotechnol. 1997, 15 (6), 542–546. 10.1038/nbt0697-542. 9181576

[ref24] Doorbar J.; Winter G. Isolation of a Peptide Antagonist to the Thrombin Receptor using Phage Display. J. Mol. Biol. 1994, 244 (4), 361–369. 10.1006/jmbi.1994.1736. 7990127

[ref25] Samli K. N.; McGuire M. J.; Newgard C. B.; Johnston S. A.; Brown K. C. Peptide-Mediated Targeting of the Islets of Langerhans. Diabetes 2005, 54 (7), 2103–2108. 10.2337/diabetes.54.7.2103. 15983211

[ref26] Fievez V.; Plapied L.; Plaideau C.; Legendre D.; des Rieux A.; Pourcelle V.; Freichels H.; Jérôme C.; Marchand J.; Préat V.; et al. *In vitro* identification of targeting ligands of human M cells by phage display. Int. J. Pharm. 2010, 394 (1), 35–42. 10.1016/j.ijpharm.2010.04.023. 20417702

[ref27] Yoo M.-K.; Kang S.-K.; Choi J.-H.; Park I.-K.; Na H.-S.; Lee H.-C.; Kim E.-B.; Lee N.-K.; Nah J.-W.; Choi Y.-J.; et al. Targeted delivery of chitosan nanoparticles to Peyer’s patch using M cell-homing peptide selected by phage display technique. Biomaterials 2010, 31 (30), 7738–7747. 10.1016/j.biomaterials.2010.06.059. 20656343

[ref28] Smart A. L.; Gaisford S.; Basit A. W. Oral peptide and protein delivery: Intestinal obstacles and commercial prospects. Expert Opin. Drug Delivery 2014, 11 (8), 1323–1335. 10.1517/17425247.2014.917077. 24816134

[ref29] Cui X.; Cao D.; Qu C.; Zhang X.; Zheng A. A study of the chemical and biological stability of vasoactive intestinal peptide. Drug Dev. Ind. Pharm. 2013, 39 (12), 1907–1910. 10.3109/03639045.2012.693503. 22670886

[ref30] aHacker D. E.; Hoinka J.; Iqbal E. S.; Przytycka T. M.; Hartman M. C. T. Highly Constrained Bicyclic Scaffolds for the Discovery of Protease-Stable Peptides via mRNA Display. ACS Chem. Biol. 2017, 12 (3), 795–804. 10.1021/acschembio.6b01006. 28146347 PMC5443354

[ref31] Góngora-Benítez M.; Tulla-Puche J.; Albericio F. Multifaceted Roles of Disulfide Bonds. Peptides as Therapeutics. Chem. Rev. 2014, 114 (2), 901–926. 10.1021/cr400031z. 24446748

[ref32] Kong X.-D.; Moriya J.; Carle V.; Pojer F.; Abriata L. A.; Deyle K.; Heinis C. De novo development of proteolytically resistant therapeutic peptides for oral administration. Nat. Biomed. Eng. 2020, 4 (5), 560–571. 10.1038/s41551-020-0556-3. 32393891

[ref33] aRozek A.; Powers J.-P. S.; Friedrich C. L.; Hancock R. E. W. Structure-Based Design of an Indolicidin Peptide Analogue with Increased Protease Stability. Biochemistry 2003, 42 (48), 14130–14138. 10.1021/bi035643g. 14640680

[ref34] aCraik D. J. Seamless Proteins Tie Up Their Loose Ends. Science 2006, 311 (5767), 1563–1564. 10.1126/science.1125248. 16543448

[ref35] aLi Y.; Li X.; Zheng X.; Tang L.; Xu W.; Gong M. Disulfide bond prolongs the half-life of therapeutic peptide-GLP-1. Peptides 2011, 32 (7), 1400–1407. 10.1016/j.peptides.2011.05.003. 21600946

[ref36] Schneider H.; Pelaseyed T.; Svensson F.; Johansson M. E. V. Study of mucin turnover in the small intestine by *in vivo* labeling. Sci. Rep. 2018, 8 (1), 5760 10.1038/s41598-018-24148-x. 29636525 PMC5893601

[ref37] de Bolos C.; Real F. X.; Lopez-Ferrer A. Regulation of mucin and glycoconjugate expression: From normal epithelium to gastric tumors. Front Biosci. 2001, 6, D1256–63. 10.2741/bolos. 11578953

[ref38] Offner G. D.; Nunes D. P.; Keates A. C.; Afdhal N. H.; Troxler R. F. The Amino-Terminal Sequence of MUC5B Contains Conserved Multifunctional D Domains: Implications for Tissue-Specific Mucin Functions. Biochem. Biophys. Res. Commun. 1998, 251 (1), 350–355. 10.1006/bbrc.1998.9469. 9790959

[ref39] Lang T. Tandem repeats structure of gel-forming mucin domains could be revealed by SMRT sequencing data. Sci. Rep. 2022, 12 (1), 20652 10.1038/s41598-022-25262-7. 36450890 PMC9712336

[ref40] Fodero L. R.; Sáez-Valero J.; Barquero M. S.; Marcos A.; McLean C. A.; Small D. H. Wheat germ agglutinin-binding glycoproteins are decreased in Alzheimer’s disease cerebrospinal fluid. J. Neurochem. 2001, 79 (5), 1022–1026. 10.1046/j.1471-4159.2001.00640.x. 11739614

[ref41] Meldrum O. W.; Yakubov G. E.; Bonilla M. R.; Deshmukh O.; McGuckin M. A.; Gidley M. J. Mucin gel assembly is controlled by a collective action of non-mucin proteins, disulfide bridges, Ca2+-mediated links, and hydrogen bonding. Sci. Rep. 2018, 8 (1), 5802 10.1038/s41598-018-24223-3. 29643478 PMC5895598

[ref42] Barmpatsalou V.; Dubbelboer I. R.; Rodler A.; Jacobson M.; Karlsson E.; Pedersen B. L.; Bergström C. A. S. Physiological properties, composition and structural profiling of porcine gastrointestinal mucus. Eur. J. Pharm. Biopharm. 2021, 169, 156–167. 10.1016/j.ejpb.2021.10.008. 34687897

[ref43] Millward S. W.; Fiacco S.; Austin R. J.; Roberts R. W. Design of Cyclic Peptides That Bind Protein Surfaces with Antibody-Like Affinity. ACS Chem. Biol. 2007, 2 (9), 625–634. 10.1021/cb7001126. 17894440 PMC3747972

[ref44] Giebel L. B.; Cass R.; Milligan D. L.; Young D.; Arze R.; Johnson C. Screening of cyclic peptide phage libraries identifies ligands that bind streptavidin with high affinities. Biochemistry 1995, 34 (47), 15430–15435. 10.1021/bi00047a006. 7492543

[ref45] Chen M.; Shi X.; Duke R. M.; Ruse C. I.; Dai N.; Taron C. H.; Samuelson J. C. An engineered high affinity Fbs1 carbohydrate binding protein for selective capture of N-glycans and N-glycopeptides. Nat. Commun. 2017, 8 (1), 15487 10.1038/ncomms15487. 28534482 PMC5457524

[ref46] Liang S.; Tang Q.; Guo X.; Li Z. A.; Guo Y.; Chang J.; Cheng B.; Song Q.; Sun J.; Dai P.; et al. Mutant glycosidases for labeling sialoglycans with high specificity and affinity. Nat. Commun. 2025, 16 (1), 1427 10.1038/s41467-025-56629-9. 39915445 PMC11802738

[ref47] Mittelstadt S. W.; Hemenway C. L.; Spruell R. D. Effects of fasting on evaluation of gastrointestinal transit with charcoal meal. J. Pharmacol. Toxicol. Methods 2005, 52 (1), 154–158. 10.1016/j.vascn.2005.04.017. 15963735

[ref48] aNguyen T.-H.; Hanley T.; Porter C. J. H.; Boyd B. J. Nanostructured liquid crystalline particles provide long duration sustained-release effect for a poorly water soluble drug after oral administration. J. Controlled Release 2011, 153 (2), 180–186. 10.1016/j.jconrel.2011.03.033. 21497623

[ref49] Cone R. A. Barrier properties of mucus. Adv. Drug Delivery Rev. 2009, 61 (2), 75–85. 10.1016/j.addr.2008.09.008. 19135107

[ref50] German J. B.; Smilowitz J. T.; Zivkovic A. M. Lipoproteins: When size really matters. Curr. Opin. Colloid Interface Sci. 2006, 11 (2–3), 171–183. 10.1016/j.cocis.2005.11.006. 20592953 PMC2893739

[ref51] Kremsmayr T.; Aljnabi A.; Blanco-Canosa J. B.; Tran H. N. T.; Emidio N. B.; Muttenthaler M. On the Utility of Chemical Strategies to Improve Peptide Gut Stability. J. Med. Chem. 2022, 65 (8), 6191–6206. 10.1021/acs.jmedchem.2c00094. 35420805 PMC9059125

[ref52] Baeriswyl V.; Heinis C. Phage selection of cyclic peptide antagonists with increased stability toward intestinal proteases. Protein Eng., Des. Sel. 2013, 26 (1), 81–89. 10.1093/protein/gzs085. 23100545

[ref53] Young T. S.; Young D. D.; Ahmad I.; Louis J. M.; Benkovic S. J.; Schultz P. G. Evolution of cyclic peptide protease inhibitors. Proc. Natl. Acad. Sci. U. S. A. 2011, 108 (27), 11052–11056. 10.1073/pnas.1108045108. 21690365 PMC3131355

[ref54] Perzborn M.; Syldatk C.; Rudat J. Enzymatical and microbial degradation of cyclic dipeptides (diketopiperazines). AMB Express 2013, 3 (1), 51 10.1186/2191-0855-3-51. 24001323 PMC3852281

[ref55] Dramsi S.; Dehoux P.; Lebrun M.; Goossens P. L.; Cossart P. Identification of four new members of the internalin multigene family of Listeria monocytogenes EGD. Infect. Immun. 1997, 65 (5), 1615–1625. 10.1128/iai.65.5.1615-1625.1997. 9125538 PMC175184

[ref56] Lecuit M.; Vandormael-Pournin S.; Lefort J.; Huerre M.; Gounon P.; Dupuy C.; Babinet C.; Cossart P. A transgenic model for listeriosis: Role of internalin in crossing the intestinal barrier. Science 2001, 292 (5522), 1722–1725. 10.1126/science.1059852. 11387478

[ref57] Macías-Rodríguez M. E.; Zagorec M.; Ascencio F.; Vázquez-Juárez R.; Rojas M. Lactobacillus fermentum BCS87 expresses mucus-and mucin-binding proteins on the cell surface. J. Appl. Microbiol. 2009, 107 (6), 1866–1874. 10.1111/j.1365-2672.2009.04368.x. 19548890

[ref58] García A.; Navarro K.; Sanhueza E.; Pineda S.; Pastene E.; Quezada M.; Henríquez K.; Karlyshev A.; Villena J.; González C. Characterization of Lactobacillus fermentum UCO-979C, a probiotic strain with a potent anti-Helicobacter pylori activity. Electronic Journal Of Biotechnology 2017, 25, 75–83. 10.1016/j.ejbt.2016.11.008.

[ref59] Brooks B. D.; Brooks A. E. Therapeutic strategies to combat antibiotic resistance. Adv. Drug Delivery Rev. 2014, 78, 14–27. 10.1016/j.addr.2014.10.027. 25450262

[ref60] Klausner E. A.; Lavy E.; Stepensky D.; Cserepes E.; Barta M.; Friedman M.; Hoffman A. Furosemide Pharmacokinetics and Pharmacodynamics following Gastroretentive Dosage Form Administration to Healthy Volunteers. Journal Of Clinical Pharmacology 2003, 43 (7), 711–720. 10.1177/0091270003254575. 12856384

[ref61] Sampathi S.; Haribhau C. J.; Kuchana V.; Junnuthula V.; Dyawanapelly S. Nanosuspension encapsulated chitosan-pectin microbeads as a novel delivery platform for enhancing oral bioavailability. Carbohydr. Polym. 2023, 319, 121177 10.1016/j.carbpol.2023.121177. 37567693

[ref62] Haber A. L.; Biton M.; Rogel N.; Herbst R. H.; Shekhar K.; Smillie C.; Burgin G.; Delorey T. M.; Howitt M. R.; Katz Y.; et al. A single-cell survey of the small intestinal epithelium. Nature 2017, 551 (7680), 333–339. 10.1038/nature24489. 29144463 PMC6022292

[ref63] Kong S.; Zhang Y. H.; Zhang W. Regulation of Intestinal Epithelial Cells Properties and Functions by Amino Acids. Biomed Res. Int. 2018, 2018, 2819154 10.1155/2018/2819154. 29854738 PMC5966675

[ref64] aKim Y. S.; Ho S. B. Intestinal goblet cells and mucins in health and disease: Recent insights and progress. Curr. Gastroenterol. Rep. 2010, 12 (5), 319–330. 10.1007/s11894-010-0131-2. 20703838 PMC2933006

[ref65] aSteer D. L.; Lew R. A.; Perlmutter P.; Smith A. I.; Aguilar M.-I. The use of β-amino acids in the design of protease and peptidase inhibitors. Letters In Peptide Science 2001, 8 (3), 241–246. 10.1023/A:1016237415473.

[ref66] aRai J. Peptide and protein mimetics by retro and retroinverso analogs. Chem. Biol. Drug Des. 2019, 93 (5), 724–736. 10.1111/cbdd.13472. 30582286

[ref67] Larnaudie S. C.; Brendel J. C.; Jolliffe K. A.; Perrier S. Cyclic peptide–polymer conjugates: Grafting-to vs grafting-from. J. Polym. Sci., Part A: polym. Chem. 2016, 54 (7), 1003–1011. 10.1002/pola.27937.

[ref68] Song Q.; Yang J.; Hall S. C. L.; Gurnani P.; Perrier S. Pyridyl Disulfide Reaction Chemistry: An Efficient Strategy toward Redox-Responsive Cyclic Peptide–Polymer Conjugates. ACS Macro Lett. 2019, 8 (10), 1347–1352. 10.1021/acsmacrolett.9b00538. 35651166

[ref69] Jeong W.-J.; Bu J.; Kubiatowicz L. J.; Chen S. S.; Kim Y.; Hong S. Peptide–nanoparticle conjugates: A next generation of diagnostic and therapeutic platforms?. Nano Convergence 2018, 5 (1), 38 10.1186/s40580-018-0170-1. 30539365 PMC6289934

[ref70] Sanna V.; Pala N.; Sechi M. Targeted therapy using nanotechnology: Focus on cancer. Int. J. Nanomed. 2014, 9, 467–483. 10.2147/IJN.S36654. PMC3896284 24531078

[ref71] Liu G.; Wang J.; Barry R.; Petersen C.; Timchalk C.; Gassman P. L.; Lin Y. Nanoparticle-based electrochemical immunosensor for the detection of phosphorylated acetylcholinesterase: An exposure biomarker of organophosphate pesticides and nerve agents. Chem.–a Eur. J. 2008, 14 (32), 9951–9959. 10.1002/chem.200800412. PMC2909471 18942695

[ref72] von Erlach T.; Saxton S.; Shi Y.; Minahan D.; Reker D.; Javid F.; Lee Y.-A. L.; Schoellhammer C.; Esfandiary T.; Cleveland C.; et al. Robotically handled whole-tissue culture system for the screening of oral drug formulations. Nat. Biomed. Eng. 2020, 4 (5), 544–559. 10.1038/s41551-020-0545-6. 32341538 PMC8357188

[ref73] Do T.; Ho F.; Heidecker B.; Witte K.; Chang L.; Lerner L. A rapid method for determining dynamic binding capacity of resins for the purification of proteins. Protein Expression Purif. 2008, 60 (2), 147–150. 10.1016/j.pep.2008.04.009. 18538581

[ref74] Evans D. F.; Pye G.; Bramley R.; Clark A. G.; Dyson T. J.; Hardcastle J. D. Measurement of gastrointestinal pH profiles in normal ambulant human subjects. Gut 1988, 29 (8), 1035 10.1136/gut.29.8.1035. 3410329 PMC1433896

[ref75] Nugent S. G.; Kumar D.; Rampton D. S.; Evans D. F. Intestinal luminal pH in inflammatory bowel disease: Possible determinants and implications for therapy with aminosalicylates and other drugs. Gut 2001, 48 (4), 571–577. 10.1136/gut.48.4.571. 11247905 PMC1728243

[ref76] Dressman J. B.; Berardi R. R.; Dermentzoglou L. C.; Russell T. L.; Schmaltz S. P.; Barnett J. L.; Jarvenpaa K. M. Upper Gastrointestinal (GI) pH in Young, Healthy Men and Women. Pharm. Res. 1990, 7 (7), 756–761. 10.1023/A:1015827908309. 2395805

[ref77] aSchuck P.; Zhao H. The role of mass transport limitation and surface heterogeneity in the biophysical characterization of macromolecular binding processes by SPR biosensing. Methods Mol. Biol. 2010, 627, 15–54. 10.1007/978-1-60761-670-2_2. 20217612 PMC4134667

[ref78] Sultana A.; Lee J. E. Measuring Protein-Protein and Protein-Nucleic Acid Interactions by Biolayer Interferometry. CP Protein Sci. 2015, 79 (1), 19–25. 10.1002/0471140864.ps1925s79. 25640894

