# Supplementary material for: Identification and Validation of Cyclic Peptides with Mucin-Selective, Location-Specific Binding in the Gastrointestinal Tract
Source: ACS Nano. 2025 Apr 11;19(15):14693–706. doi: 10.1021/acsnano.4c13520 (PMC12020424; doi:10.1021/acsnano.4c13520)
Supplement: Supplementary file 1 — nn4c13520_si_001.pdf [file nn4c13520_si_001.pdf]

Identification and validation of cyclic peptides with mucin-selective, location-specific binding in the gastrointestinal tract

Deepak A. Subramanian[1,2], Austin Chin[1], Shi Yunhua[2], Gary W. Liu[2], Robert Langer[1,2,3], Giovanni Traverso[2,3,4,]*

[1]Department of Chemical Engineering, Massachusetts Institute of Technology, Cambridge, MA 02139, USA.

[2]David H. Koch Institute for Integrative Cancer Research, Massachusetts Institute of Technology, Cambridge, MA 02139, USA.

[3] Department of Mechanical Engineering, Massachusetts Institute of Technology, Cambridge, MA 02139, USA.

[4] Division of Gastroenterology, Brigham and Women's Hospital, Harvard Medical School, Boston, MA 02115, USA.

[*]Corresponding author. Email: cgt20@mit.edu

Supporting Information

Table S1. Mucin-binding kinetic constants and dissociation constant for peptide “hits”.

Peptide	Mucin/pH	k_{on} ($\frac{1}{M\ s}$)	k_{off} ($\frac{1}{s}$)	k_d (M)
**M2.4**	**MUC2/7.2**	$\mathbf{5.77 \times 10^{4}}$	$\mathbf{6.47 \times 10^{-3}}$	$\mathbf{1.12 \times 10^{-7}}$
**M2.4**	**MUC2/3.35**	$\mathbf{1.05 \times 10^{5}}$	$\mathbf{4.91 \times 10^{-3}}$	$\mathbf{4.66 \times 10^{-8}}$
M2.4	MUC5AC/1.82	2.95×10^{4}	1.10×10^{-3}	3.72×10^{-8}
**M2.3**	**MUC2/7.2**	$\mathbf{6.99 \times 10^{5}}$	$\mathbf{4.97 \times 10^{-3}}$	$\mathbf{7.11 \times 10^{-9}}$
**M2.3**	**MUC2/3.35**	$\mathbf{6.21 \times 10^{6}}$	$\mathbf{1.58 \times 10^{-3}}$	$\mathbf{2.55 \times 10^{-10}}$
M2.3	MUC5AC/1.82	2.70×10^{4}	1.77×10^{-3}	6.54×10^{-8}
M5.2	MUC2/7.2	3.99×10^{4}	4.48×10^{-3}	1.12×10^{-7}
M5.2	MUC2/3.35	3.27×10^{4}	9.13×10^{-3}	2.79×10^{-7}
**M5.2**	**MUC5AC/1.82**	$\mathbf{4.03 \times 10^{5}}$	$\mathbf{3.17 \times 10^{-3}}$	$\mathbf{7.88 \times 10^{-9}}$
M5.3	MUC2/7.2	6.33×10^{4}	4.73×10^{-3}	7.48×10^{-8}
M5.3	MUC2/3.35	7.68×10^{4}	5.07×10^{-3}	6.60×10^{-8}
**M5.3**	**MUC5AC/1.82**	$\mathbf{1.01 \times 10^{6}}$	$\mathbf{1.55 \times 10^{-3}}$	$\mathbf{1.54 \times 10^{-9}}$

Table S2. Fluorescence counts for *ex vivo* binding of MUC2-selective peptides to the small intestine.

Replicate	M2.1	M2.2	M2.3	M2.4	WGA	Free dye
1	4.00E+09	2.52E+09	2.03E+09	8.53E+09	6.39E+09	1.14E+08
2	1.24E+10	9.14E+09	1.94E+10	1.68E+10	1.34E+10	8.24E+09
3	5.38E+09	5.14E+09	8.02E+09	8.99E+09	9.58E+09	4.20E+09

Table S3. Fluorescence counts for *ex vivo* binding of MUC2-selective peptides to the stomach.

Replicate	M2.1	M2.2	M2.3	M2.4	WGA	Free dye
1	9.60E+09	9.39E+09	1.94E+10	1.22E+10	2.76E+10	1.56E+10
2	9.70E+09	5.33E+09	2.36E+09	5.43E+09	1.09E+10	3.06E+09
3	4.76E+09	4.99E+09	3.74E+09	4.84E+09	6.69E+09	3.59E+09

Table S4. Fluorescence counts for *ex vivo* binding of MUC5AC-selective peptides to the small intestine.

Replicate	M5.1	M5.2	M5.3	M5.4	WGA	Free dye
1	1.14E+10	1.60E+10	4.80E+09	1.18E+10	1.78E+10	1.29E+10
2	1.55E+10	1.06E+10	5.07E+09	5.52E+09	1.58E+10	1.15E+10
3	1.55E+10	1.12E+10	4.36E+09	5.88E+09	1.36E+10	7.09E+09

Table S5. Fluorescence counts for *ex vivo* binding of MUC5AC-selective peptides to the stomach.

Replicate	M5.1	M5.2	M5.3	M5.4	WGA	Free dye
1	8.76E+09	9.83E+09	3.55E+09	3.53E+09	6.93E+09	3.13E+09
2	7.25E+09	7.45E+09	7.16E+09	6.48E+09	1.15E+10	4.22E+09
3	2.10E+10	1.28E+10	2.56E+10	1.56E+10	3.49E+10	6.83E+09

Table S6. Overall nanoparticle "score" and selectivity values for binding of MUC2-selective peptides to the small intestine.

Metric	M2.1	M2.2	M2.3	M2.4	WGA	Free dye
"Score"	0.550	0.243	1.056	1.294	1	0
Selectivity	0.298	0.396	0.925	0.741	0.333	0

Table S7. Overall nanoparticle "score" and selectivity values for binding of MUC5AC-selective peptides to the stomach.

Metric	M5.1	M5.2	M5.3	M5.4	WGA	Free dye
"Score"	0.800	0.805	0.393	0.242	1	0
Selectivity	0.370	0.604	1	0.920	0.333	0

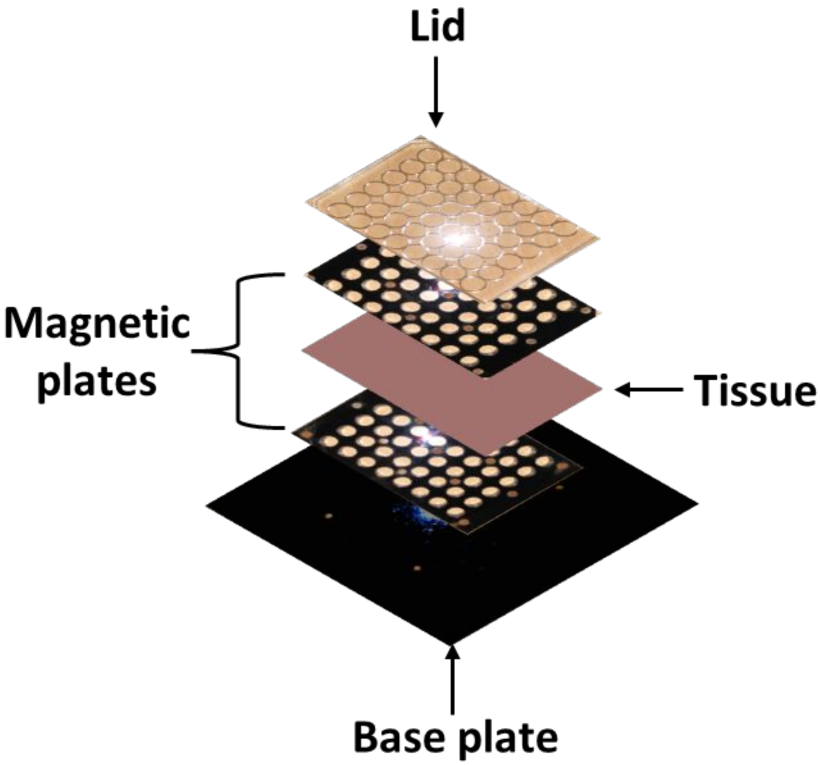

Figure S1. Mounted setup for *ex vivo* tissue binding. The setup consists of four parts. The bottom plate is a black polystyrene plate (20 cm x 20 cm), which serves to minimize background fluorescence during imaging studies. The next two plates are magnetic plates with 48 wells cut into the plates, which can suspend a piece of tissue through the magnetic force sticking the plates together. The luminal side of the tissue is presented upwards, allowing for incubation of mucin-binding formulations. The final plate is a standard 48-well plate cover, which is used to enclose the tissue setup.

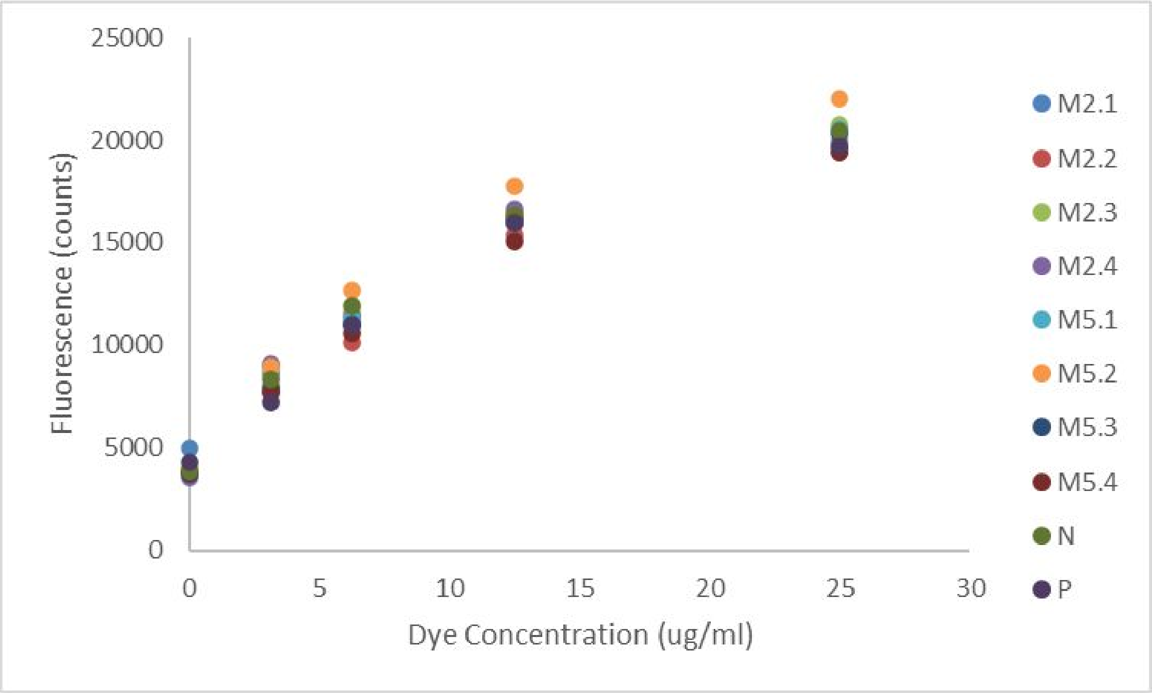

Figure S2. Comparison of fluorescence of peptide-AF647, free AF647, and WGA-AF647 conjugates at various concentrations. Here, drug concentration refers to the concentration of the AF647 dye.

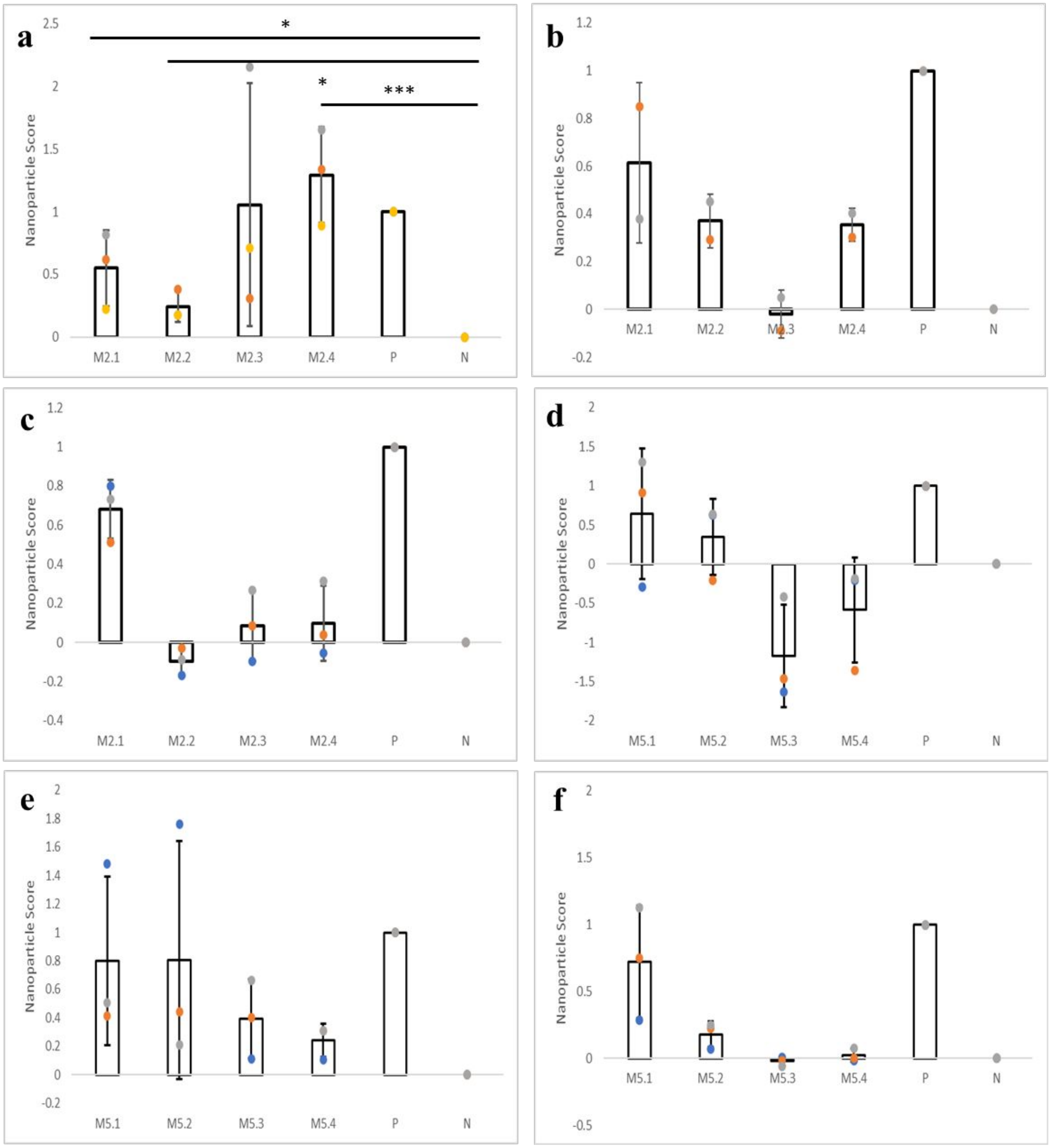

Figure S3. *Ex vivo* binding score results for MUC2-selective peptide hits to the small intestine (a), stomach (b), and esophagus (c), and MUC5AC-selective peptide hits to the small intestine (d), stomach (e), and esophagus (f). Bars show average of individual experiments (n=3), and error bars show standard deviation. Individual data points are also indicated on the graphs.

Values on the y-axis correspond to the nanoparticle score, which is defined as $\boldsymbol{Score_i} = \frac{f_i - f_n}{f_p - f_n}$.
Statistical significance is shown by: * - $p < 0.05$, *** - $p < 0.005$.

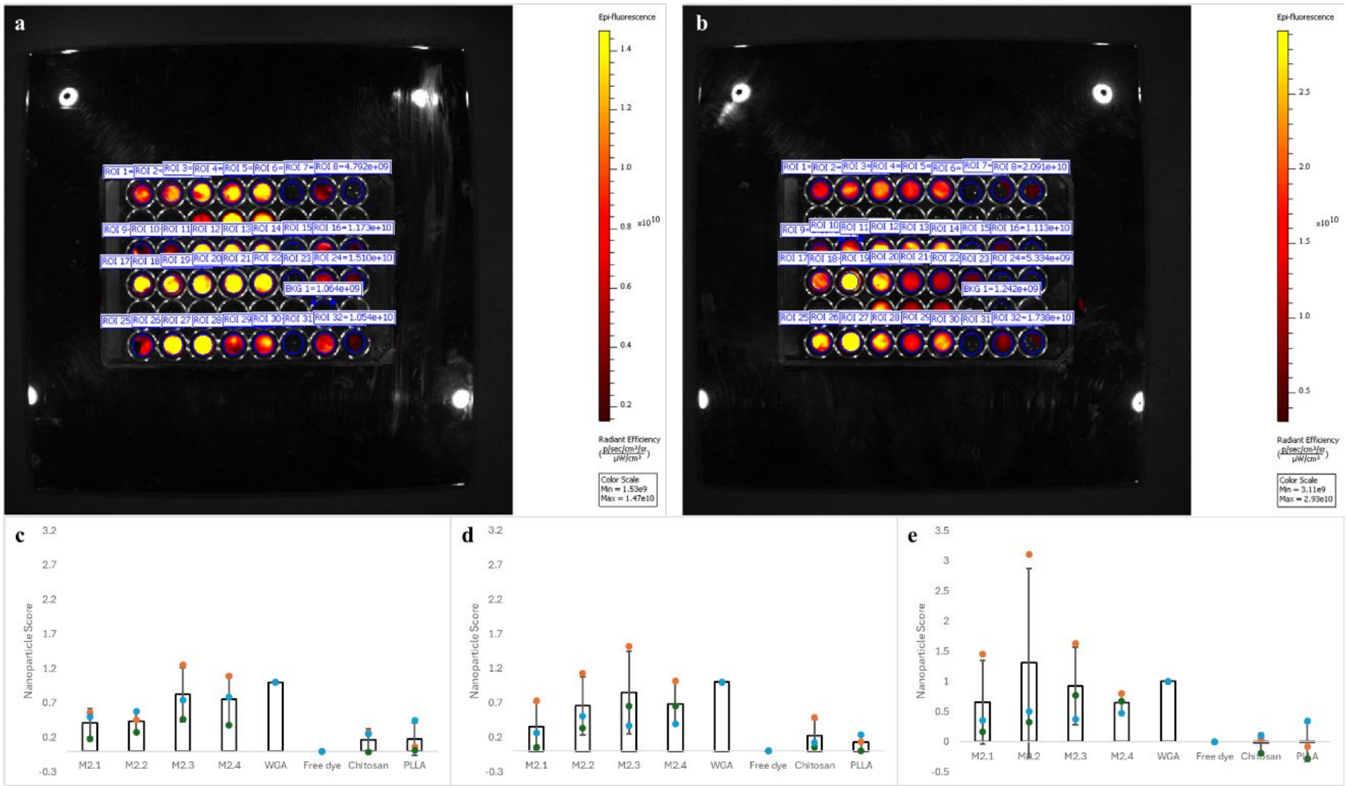

Figure S4. *Ex vivo* binding study for MUC2-selective peptide to the small intestine at various time points. Representative IVIS images are shown at 2 and 4 hours (a) and 8 hours (b); the top half of the IVIS image in (a) represents the 2 hour time point, while the bottom half of the image in (a) represents the 4 hour time point. The columns (from left to right) represent M2.1, M2.2, M2.3, M2.4, WGA-AF647, AF647, chitosan-Cy5, and poly(D,L-lactic acid)-Cy5. Nanoparticle score results are shown for each peptide/control at 2 hours (c), 4 hours (d), and 8 hours (e). The nanoparticle score (on the y-axis) is defined as $\boldsymbol{Score_i} = \frac{f_i - f_n}{f_p - f_n}$.

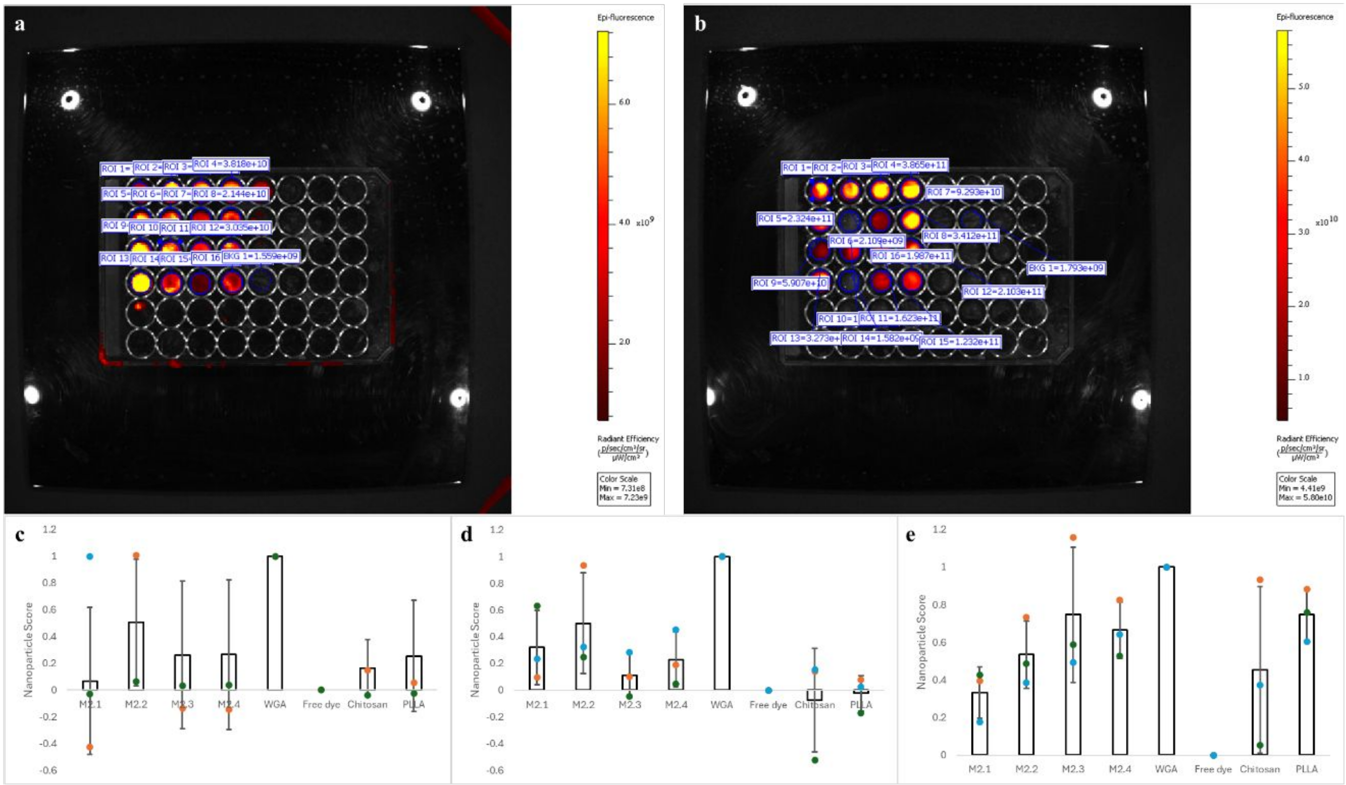

Figure S5. *Ex vivo* binding study for MUC2-selective peptide to the stomach at various time points. Representative IVIS images are shown at 2 and 4 hours (a) and 8 hours (b); the top half of the IVIS image in (a) represents the 2 hour time point, while the bottom half of the image in (a) represents the 4 hour time point. The columns (from left to right) represent M2.1, M2.2, M2.3, M2.4 (row 1), WGA-AF647, AF647, chitosan-Cy5, and poly(D,L-lactic acid)-Cy5 (row 2). Nanoparticle score results are shown for each peptide/control at 2 hours (c), 4 hours (d), and 8 hours (e). The nanoparticle score (on the y-axis) is defined as $\boldsymbol{Score_i} = \frac{f_i - f_n}{f_p - f_n}$.

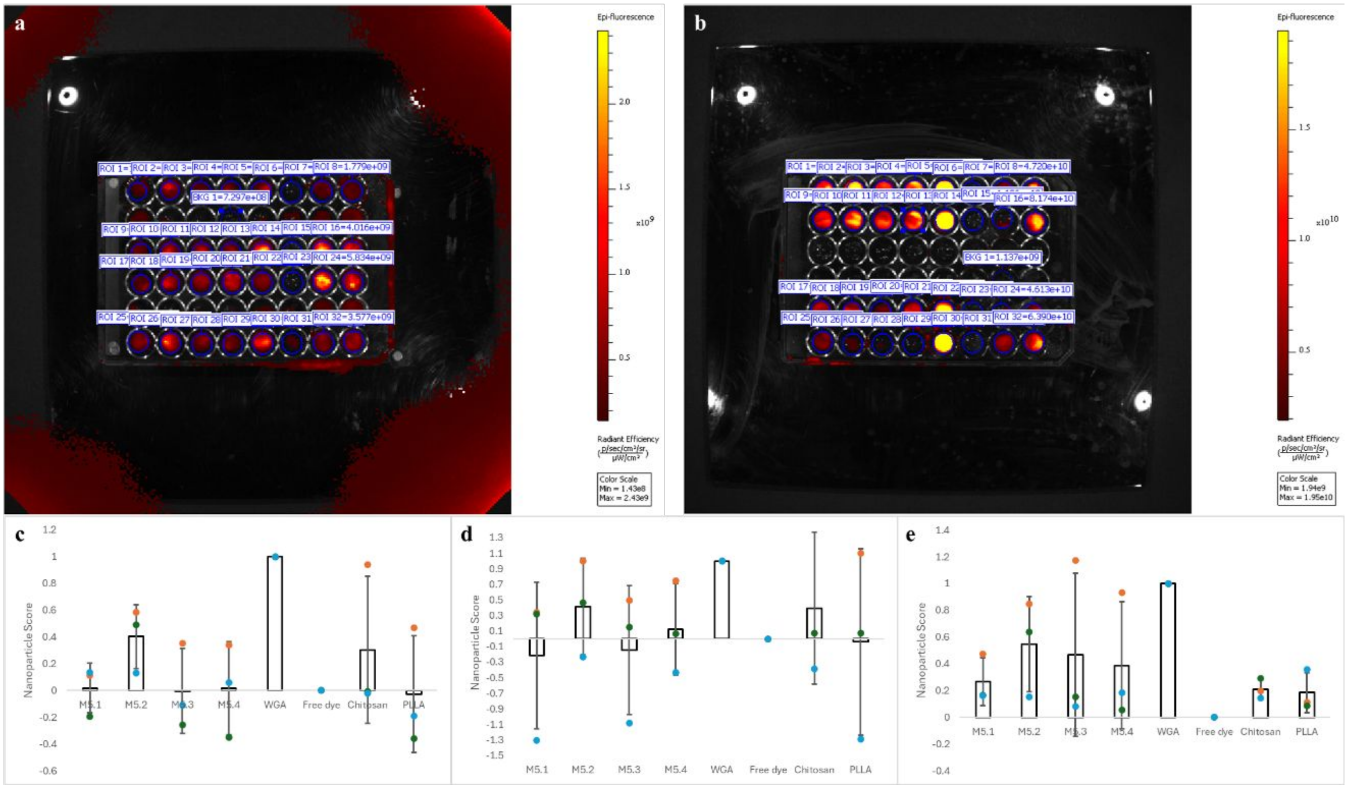

Figure S6. *Ex vivo* binding study for MUC5AC-selective peptide to the small intestine at various time points. Representative IVIS images are shown at 2 and 4 hours (a) and 8 hours (b); the top half of the IVIS image in (a) represents the 2 hour time point, while the bottom half of the image in (a) represents the 4 hour time point. The columns (from left to right) represent M5.1, M5.2, M5.3, M5.4, WGA-AF647, AF647, chitosan-Cy5, and poly(D,L-lactic acid)-Cy5. Nanoparticle score results are shown for each peptide/control at 2 hours (c), 4 hours (d), and 8 hours (e). The nanoparticle score (on the y-axis) is defined as $\boldsymbol{Score_i} = \frac{f_i - f_n}{f_p - f_n}$.

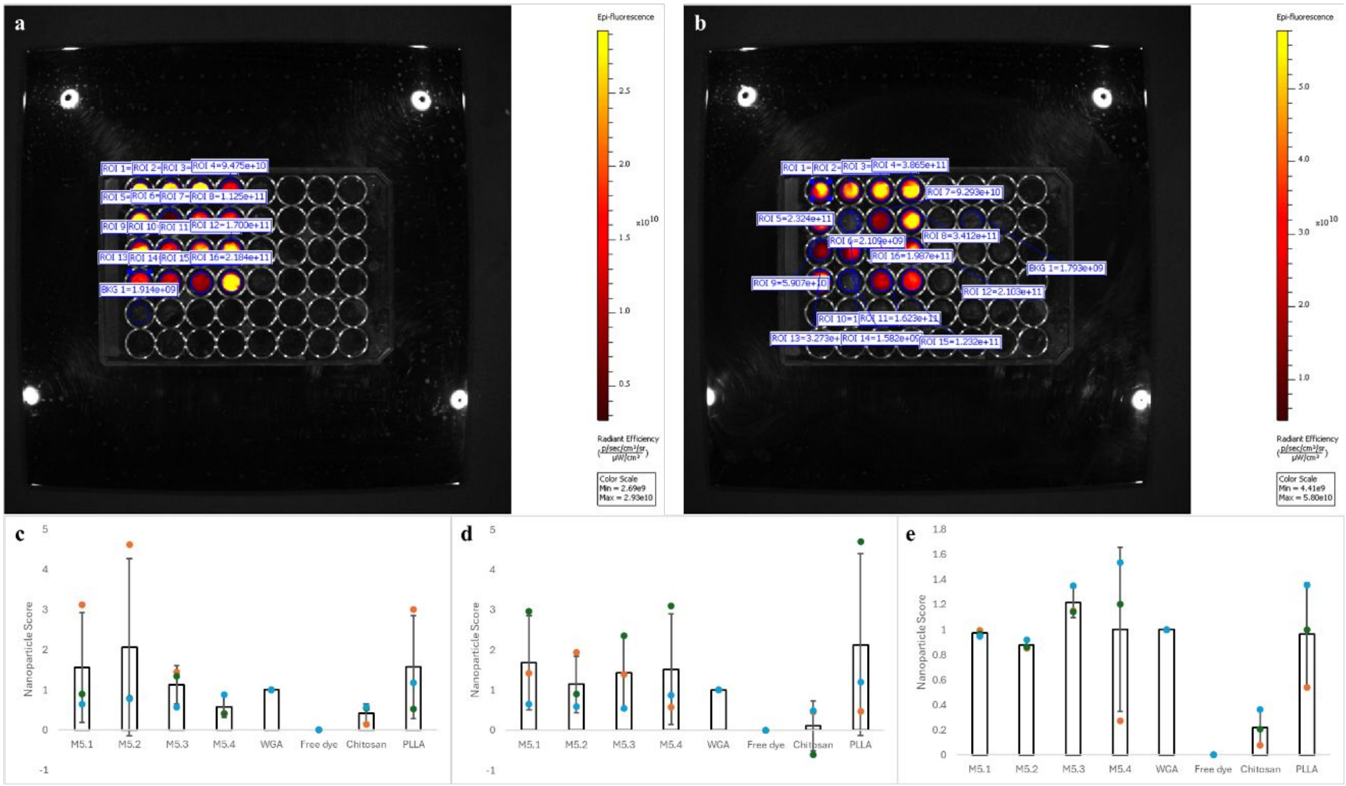

Figure S7. *Ex vivo* binding study for MUC5AC-selective peptide to the stomach at various time points. Representative IVIS images are shown at 2 and 4 hours (a) and 8 hours (b); the top half of the IVIS image in (a) represents the 2 hour time point, while the bottom half of the image in (a) represents the 4 hour time point. The columns (from left to right) represent M5.1, M5.2, M5.3, M5.4 (row 1), WGA-AF647, AF647, chitosan-Cy5, and poly(D,L-lactic acid)-Cy5 (row 2). Nanoparticle score results are shown for each peptide/control at 2 hours (c), 4 hours (d), and 8 hours (e). The nanoparticle score (on the y-axis) is defined as $\boldsymbol{Score_i} = \frac{f_i - f_n}{f_p - f_n}$.

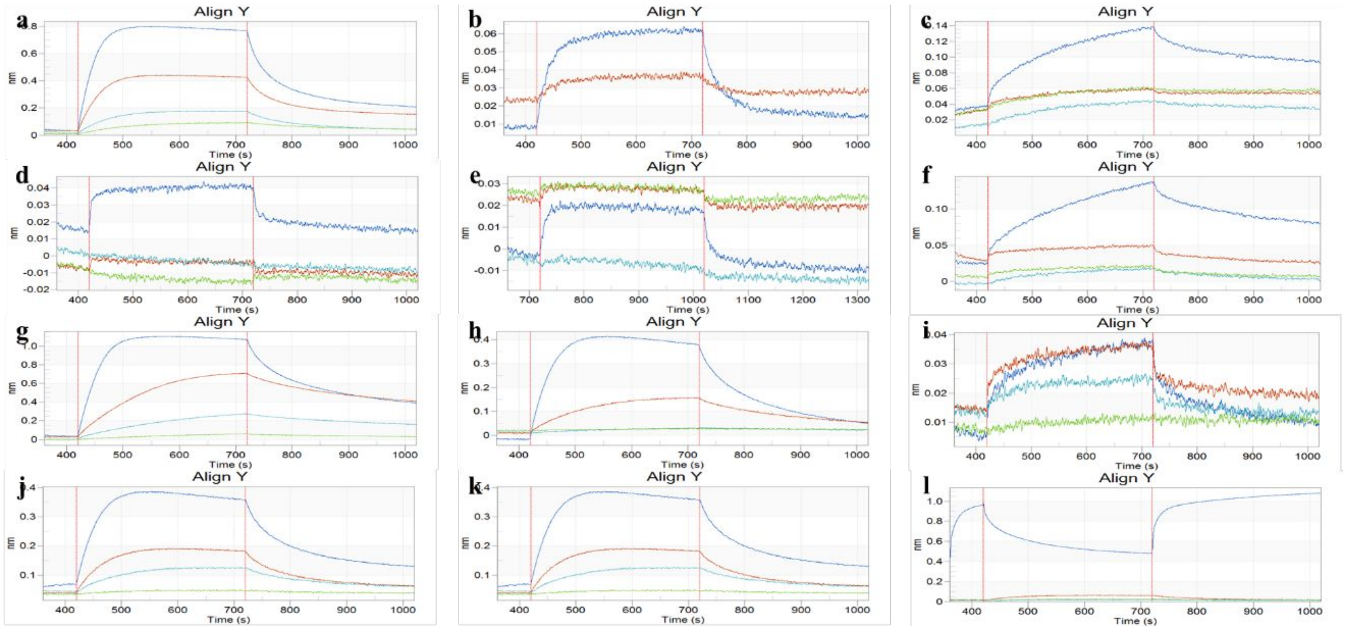

Figure S8. Association and dissociation curves measured by bio-layer interferometry. Results shown are for hit CASSLSVRC to MUC2 at pH 7.2 (a), MUC2 at pH 3.35 (b), and MUC5AC at pH 1.82 (c), hit CDGRPDRAC to MUC2 at pH 7.2 (d), MUC2 at pH 3.35 (e), and MUC5AC at pH 1.82 (f), hit CGPIYTALC to MUC2 at pH 7.2 (g), MUC2 at pH 3.35 (h), and MUC5AC at pH 1.82 (i), and hit CTALTMGMC to MUC2 at pH 7.2 (j), MUC2 at pH 3.35 (k), and MUC5AC at pH 1.82 (l).

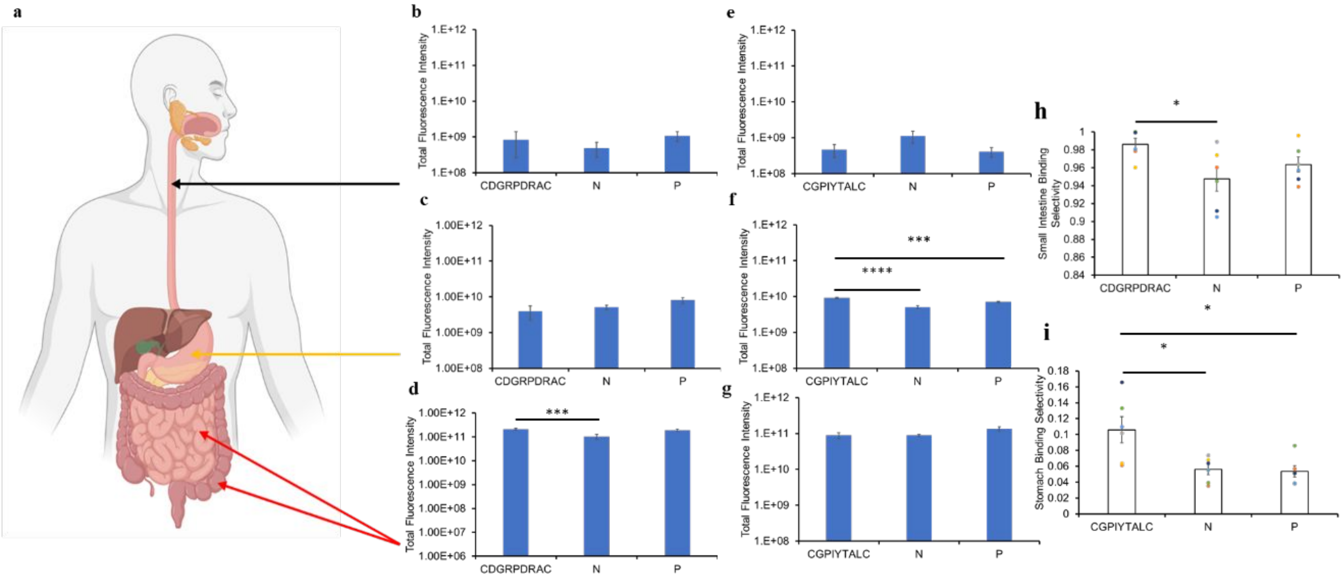

Figure S9. Detailed *in vivo* distribution results across different regions of the GI tract (a), showing the MUC2-binding peptide hit fluorescence in the esophagus (b), MUC2-binding peptide hit fluorescence in the stomach (c), MUC2-binding peptide hit fluorescence in the small intestine/colon (d), MUC5AC-binding peptide hit fluorescence in the esophagus (e), MUC5AC-binding peptide hit fluorescence in the stomach (f), and MUC5AC-binding peptide hit fluorescence in the small intestine/colon (g). Detailed selectivity results are shown for MUC2-selective (h) and MUC5AC-selective (i) hits. Error bars represent standard error of measurement. * - $p < 0.05$, *** - $p < 0.005$.
